# High-Throughput MicroRNA (miRNAs) Arrays Unravel the Prognostic Role of MiR-211 in Pancreatic Cancer

**DOI:** 10.1371/journal.pone.0049145

**Published:** 2012-11-14

**Authors:** Elisa Giovannetti, Arjan van der Velde, Niccola Funel, Enrico Vasile, Vittorio Perrone, Leticia G. Leon, Nelide De Lio, Amir Avan, Sara Caponi, Luca E. Pollina, Valentina Gallá, Hiroko Sudo, Alfredo Falcone, Daniela Campani, Ugo Boggi, Godefridus J. Peters

**Affiliations:** 1 Department of Medical Oncology, VU University Medical Center, Amsterdam, The Netherlands; 2 Centre Integrative Bioinformatics, VU University, Amsterdam, The Netherlands; 3 Department of Surgical Pathology, University of Pisa, Pisa, Italy; 4 Azienda Ospedaliera Universitaria Pisana, Istituto Tumori Toscano, Pisa, Italy; 5 Department of General Surgery and Transplants, University of Pisa, Pisa, Italy; 6 New Frontiers Research Laboratories, Toray Industries Inc., Kanagawa, Japan; University of Illinois at Chicago, United States of America

## Abstract

**Background:**

Only a subset of radically resected pancreatic ductal adenocarcinoma (PDAC) patients benefit from chemotherapy, and identification of prognostic factors is warranted. Recently miRNAs emerged as diagnostic biomarkers and innovative therapeutic targets, while high-throughput arrays are opening new opportunities to evaluate whether they can predict clinical outcome. The present study evaluated whether comprehensive miRNA expression profiling correlated with overall survival (OS) in resected PDAC patients.

**Methodology/Principal Findings:**

High-resolution miRNA profiles were obtained with the Toray's *3D-Gene*™-miRNA-chip, detecting more than 1200 human miRNAs. RNA was successfully isolated from paraffin-embedded primary tumors of 19 out of 26 stage-pT3N1 homogeneously treated patients (adjuvant gemcitabine 1000 mg/m^2^/day, days-1/8/15, every 28days), carefully selected according to their outcome (OS<12 (N = 13) vs. OS>30 months (N = 6), i.e. short/long-OS). Highly stringent statistics included t-test, distance matrix with Spearman-ranked correlation, and iterative approaches. Unsupervised hierarchical analysis revealed that PDACs clustered according to their short/long-OS classification, while the feature selection algorithm RELIEF identified the top 4 discriminating miRNAs between the two groups. These miRNAs target more than 1500 transcripts, including 169 targeted by two or more. MiR-211 emerged as the best discriminating miRNA, with significantly higher expression in long- vs. short-OS patients. The expression of this miRNA was subsequently assessed by quantitative-PCR in an independent cohort of laser-microdissected PDACs from 60 resected patients treated with the same gemcitabine regimen. Patients with low miR-211 expression according to median value had a significantly shorter median OS (14.8, 95%CI = 13.1–16.5, vs. 25.7 months, 95%CI = 16.2–35.1, log-rank-P = 0.004). Multivariate analysis demonstrated that low miR-211 expression was an independent factor of poor prognosis (hazard ratio 2.3, P = 0.03) after adjusting for all the factors influencing outcome.

**Conclusions/Significance:**

Through comprehensive microarray analysis and PCR validation we identified miR-211 as a prognostic factor in resected PDAC. These results prompt further prospective studies and research on the biological role of miR-211 in PDAC.

## Introduction

With a 5-year survival rate of less than 5%, pancreatic ductal adenocarcinoma (PDAC), including more than 90% of pancreatic cancers, is the most lethal among the major solid tumors [1]. In recent years, there have been important advances in the understanding of molecular biology of pancreatic cancer, as well as in diagnosis and staging. However, minimal progress has been achieved in prevention, early diagnosis, treatment and outcomes [2].

Surgical resection is the only curative modality for PDAC, but only 15–20% of patients have resectable disease at the time of diagnosis. Nevertheless, the prognosis of patients after complete resection is poor, with 3-year disease-free survival (DFS) rate at 27% (95% confidence interval (CI): 23–32%) and median overall survival (OS) of 15–19 months [3].

Only a subset of radically resected PDAC patients benefit from chemotherapy, and adjuvant treatments can have substantial toxicities [4]. Therefore, novel biomarkers of sensitivity to adjuvant therapy are urgently warranted in order to individualize clinical management and improve therapeutic outcome [5].

Extensive studies have characterized the complex genetic networks and transcriptomics alterations underlying the development and progression of PDAC [6]. The recent discovery of microRNAs (miRNAs) has provided additional insights potentially explaining the gap that exists between tumor genotype and phenotype.

MiRNA are a class of small non-coding evolutionarily conserved RNAs [19]–[23 nucleotides] that have been found in animal and plant cells. As of today, 1921 unique mature human miRNAs are listed in the miRBase database (Release 18, November 2011) [7]. MicroRNA genes are transcribed as non-coding transcripts, and are processed through a series of sequential steps involving the RNase III enzymes, Drosha and Dicer. The processed microRNAs are finally incorporated into the RNA-induced silencing complex (RISC) to direct this complex to down-regulate gene expression via binding to the 3′UTR of the target mRNAs. Plant and some animal miRNAs form perfect base pairs with their target mRNAs, resulting in their degradation. However, most of the human miRNAs bind to their target 3′UTRs with imperfect complementarities and therefore induce translational repression [8].

The pivotal regulatory role of each miRNA in controlling expression of multiple gene transcripts offers a unique opportunity of identifying critical miRNAs as informative biomarkers for detection, diagnosis and prognosis of tumors that result from deregulation of multiple genes [9]. This underlying biological mechanism was most likely the reason why expression patterns of 217 miRNAs were found to classify cancer types more accurately than the information based on expression profile of ∼16000 mRNAs [10].

The role of miRNAs in the control of proliferation/differentiation and apoptosis, and their aberrant expression in many tumors, indicated that they might function as tumor suppressors and oncogenes, suggesting their use for diagnostic and therapeutic purposes. Furthermore, selected miRNAs may influence tumor malignant behavior and response to chemotherapy [11].

Our previous studies focusing on miR-21 showed that both Caucasian and Asian patients harboring high expression of this miRNA in their PDAC specimens had a significantly shorter survival [12], [13]. This miRNA has been referred to as an “oncomir” (i.e. a miRNA with oncogenic properties) because it is almost omnipresent and overexpressed in human tumors. Recent *in vivo* studies in miR-21 overexpressing mice model established by Cre/Tet-off technologies, demonstrated its oncogenic role, showing its significant impact on tumor initiation, maintenance, survival and invasion [14].

However, high-throughput technological innovations in detecting hundreds of microRNAs provide new effective ways to unravel the role of other key miRNAs regulating multiple genes that might explain why patients with similar clinicopathological characteristics can have considerable variation in clinical outcomes. Therefore, in the present study we evaluated whether comprehensive miRNA expression profiling, using a miRNA chip detecting more than 1200 types of human miRNA, can distinguish between PDAC patients with very short OS compared to long-term survivors.

In particular, we carefully selected 26 PDAC patients with homogeneous clinicopathological characteristics who underwent resection with curative intent and were treated with three cycles of standard gemcitabine adjuvant regimen. Half of these patients had a dismal prognosis, dying within 1 year of diagnosis, whereas the other 13 patients survived more than 30 months. The miRNA microarray analysis was performed in 19 samples that passed the RNA quality criterion, including 13 patients with short survival and 6 patients with long survival. Since miR-211 expression status emerged as the most predictive biomarker for treatment outcome in these patients, further analysis of miR-211 expression was performed in a second cohort of 60 patients, all treated with the same adjuvant therapy. This independent set confirmed the significant association of miR-211 expression status with both OS and DFS.

## Methods

### Patients

Patients who underwent radical surgical resection with curative intent (pancreatico-duodenectomy, total pancreatectomy and distal pancreatectomy) at the Department of General Surgery and Transplant, University Hospital of Pisa (Pisa, Italy), between 2000 and 2010 were retrospectively reviewed using electronic medical records. Among them, for the high-resolution miRNA expression profiling we selected 26 patients with similar pathological findings, clinical characteristics, and treatment but considerable variation in clinical outcomes. In particular, half of these patients had an extremely poor prognosis, dying within 1 year of diagnosis and were classified as “short-OS”, whereas the other 13 patients survived more than 30 months, and were classified as “long-OS”. The characteristics of these 2 groups are reported in Table 1.

**Table 1:** Clinical characteristics of the PDAC patients.

Characteristic	Patients for the miRNA profiling n (%)		Patients of the validation cohort n (%)
	Short-OS	Long-OS	
*No. Patients*	13	13	60
*Age. median [range]*	64 (37–71)	63 (56–79)	
≤65	8 (61.5)	6 (46.2)	36 (60.0)
>65	5 (38.5)	7 (53.8)	24 (40.0)
*Sex*			
Male	7 (53.8)	3 (23.1)	28 (46.7)
Female	6 (46.2)	10 (76.9)	32 (53.3)
*Operation procedure*			
pancreatico-duodenectomy	11 (84.6)	9 (69.2)	46 (76.7)
total pancreatectomy	0 (0.0)	0 (0.0)	3 (5.0)
distal pancreatectomy	2 (15.4)	4 (30.8)	11 (18.3)
*TNM Stage*			
pT3 N0 Mx	0 (0.0)	1 (7.7)	0 (0.0)
pT3 N1 Mx	13 (100.0)	12 (92.3)	60 (100.0)
*Nodal status*			
N0	0 (0.0)	1 (7.7)	0 (0.0)
N1	13 (100.0)	12 (92.3)	60 (100.0)
*Grading*			
G1	0 (0.0)	0 (0.0)	3 (5.00)
G2	8 (61.5)	9 (69.2)	27 (45.0)
G3	5 (38.5)	4 (30.8)	30 (50.0)
Unknown	0 (0.0)	0 (0.0)	0 (0.0)
*Resection margins*			
R0	12 (92.2)	12 (92.2)	54 (90.0)
R1	1 (7.8)	1 (7.8)	6 (10.0)
*Vascular invasion*			
Yes	4 (30.8)	3 (23.1)	23 (38.3)
No	9 (69.2)	10 (76.9)	37 (31.7)
*Perineural invasion*			
Yes	7 (53.8)	9 (69.2)	31 (51.7)
No	6 (46.2)	4 (30.8)	29 (48.3)

The validation cohort was composed of other 60 radically resected PDAC patients diagnosed and treated in the same period, with their characteristics also described in the Table 1. All these patients underwent gemcitabine-based adjuvant treatment, as described previously [15].

### Ethics

All the patients gave their written informed consent to the sample collection and analysis, and the study has received approval from the Ethics committee of Pisa University Hospital as a follow‐up study of the research protocol entitled “Pharmacogenetics of gemcitabine‐related genes in pancreas cancer: correlation with clinical outcome and tolerability” [15]. The responsible investigators ensure that this study was conducted according to the Declaration of Helsinki, the European Guidelines on Good Clinical Practice, and relevant national and regional authority requirements.

### Tissues

Formalin Fixed Paraffin Embedded (FFPE) sections were carefully reviewed for diagnosis and tumor content. Because of the long experience of our pathology laboratory on large cohorts of radically resected PDAC patients, there was no difficulty in selecting areas with morphological defined cancer cells [16]. The tumors were classified and evaluated for tumor staging and grading as proposed by the WHO, as reported in Table 1.

### RNA extraction from FFPE slides

Histological sections (10 µm) were prepared from each FFPE specimen. Paraffin was removed by xylene treatment and tissues were washed with ethanol twice to remove xylene. Tissues were then treated with proteinase K at 37°C overnight. Following centrifugation, the supernatant was processed with a silica-based spin column (New Frontiers Research Laboratories, Toray Industries Inc., Kanagawa, Japan) in order to obtain purified total RNA. The degrees of RNA cross-linking and RNA degradation were analyzed by electrophoresis using an Agilent 2100 Bioanalyzer (Agilent Technologies, Santa Clara, CA, USA). The samples that showed the majority of RNAs at >4,000 nucleotides due to cross-linking, or the majority of RNAs at <1,000 nucleotides due to degradation in the electrophoresis patterns were unsuitable for the miRNA analysis and thus not used. Of the 26 studied samples, 19 samples passed this criterion and were used in the miRNA profiling.

For the 60 samples used as an independent validation set, a mean of 5000 neoplastic cells were then dissected using the Leica LMD6500 instrument (Leica, Wetzlar, Germany), as described previously [17]. The precision of the narrow focus of the laser beam resulted in the capture of individual cells with high degree of accuracy (Figure S1). RNA was successfully isolated using the RecoverAll Total Nucleic Acid Isolation kit (Ambion, Applied Biosystems, Foster City, CA, USA), according to the manufacturer's instructions. RNA yields and purity were checked at 260 and 280 nm with NanoDrop®-1000 Detector (NanoDrop-Technologies, Wilmington, USA).

### MiRNA profiling

We utilized Toray's 3D-Gene™ (Toray Industries, Japan) human microRNA chips for miRNA expression profiling. The reproducibility and comparability to Taqman RT-PCR, and the experimental procedures of Toray's microarray, were described previously [18], [19]. Briefly, 500 ng total RNA extracted from FFPE section was analyzed for miRNA profiling using microarray, 3D-Gene® miRNA oligo chip v.16 (Toray Industries) according to the manufacturer's protocol vE1.10. The number of mounted miRNAs on this microarray is 1212 in total. Microarray was scanned and the obtained images were numerated using 3D-Gene® scanner 3000 (Toray Industries). The expression level of each miRNA was globally normalized using the background-subtracted signal intensity of the entire miRNAs in each microarray (Description S1).

All microarray data from this study are in agreement with Minimum Information About a Microarray Experiment (MIAME) and publicly available through the NCBI's Gene Expression Omnibus (GEO) database (http://www.ncbi.nlm.nih.gov/projects/geo/) under the series record GSE38781.

### Overall clustering

To explore differences in expression patterns between the two groups of samples, we selected miRNAs that showed a significant difference in expression. The t-test was performed on the long-OS and short-OS groups for all miRNAs and the ones that did not appear to be significantly different (p>0.05) have been filtered out. Hierarchical unsupervised cluster analysis was performed on the remaining 170 miRNAs. Two-tailed Spearman ranked correlation was used to generate a distance matrix. Subsequently the distance matrix was used to generate clusters for both miRNAs and samples using a hierarchical clustering algorithm, based on average linkage [20]–[21].

### Analysis with RELIEF and iterative RELIEF

A feature selection algorithm, RELIEF [22]–[24], was employed on the complete data set in order to discover the most discriminating miRNAs. RELIEF is an iterative algorithm that assigns weights to features (i.e., miRNA expression values) according to distances between features within and among groups. Out of the 1212 miRs, 703 miRs that have a maximum of one missing value over all samples were selected. The RELIEF algorithm was applied in order to select the top 10 highest weighing miRNAs. In a following analysis we generated 100 random sets of 6 out of 13 samples classified as short. On each random set of samples, combined with the 6 samples classified as long the RELIEF algorithm was applied to select the top 10 highest weighing miRs. For all miRNAs appearing in the top 10 a score was kept and the top 10 most appearing miRNAs were selected.

### Top miRNAs target genes

A search was performed on the predicted targets for the most discriminating miRNAs identified in our study using the *TargetScan* web interface v.6.1 (http://www.targetscan.org/) and miRDB version 4.0 (http://mirdb.org/miRDB/index.html). Following comparison of all datasets, a subset of genes that were targeted by more than one miRNA was generated.

### Reverse transcription (RT) and quantitative-PCR analysis of miR-211 and miR-4321

In order to validate the findings of the microarray analysis, we evaluated the expression of the most discriminating miRNA, miR-211, as well as of the rarely investigated miR-4321, in an independent cohort of PDAC patients. RNA (10–100 ng) was reverse transcribed and the resulting cDNA was amplified using the specific custom TaqMan®-MicroRNA-assays (Applied Biosystems) for miR-211 and miR-4321. We performed a preliminary analysis of 3 endogenous controls (RNU1, RNU6 and RNU43) in a series of 10 PDAC cells. Since the values of RNU6 were the closest to the geometric mean values of these genes, we used this housekeeping for the normalization of all the following analysis. The PCR reactions were performed in the 7500HT sequence detection system (Applied Biosystems), in accordance with the manufacturer's instructions. Specimens were amplified in duplicate with appropriate non-template controls. Amplification data were normalized to RNU6 expression. Quantification of relative expression (reported as arbitrary units [a.u.]) was performed using the ΔCt method. Quantitative-PCR data showed a variability coefficient of Ct always lower than 2% of mean values.

### Correlation of miR-211 and miR-4321 with outcome

Comparison of clinical information and miRNA expression levels were made using Pearson χ^2^ test and Wilcoxon test. The relationship between miRNA expression and outcome was evaluated by stratifying the patients with respect to the median expression value (high versus low expression). The analyses of the samples were done in a blinded fashion relative to clinical outcome.

OS was calculated from the date of surgery to the date of death, DFS was defined as the time from the date of surgery to the date of first relapse or death. Survival curves were constructed using the Kaplan-Meier method, and differences were analyzed using log-rank test. The significant prognostic variables of OS and DFS in univariate analysis were included in multivariate analyses, using Cox's proportional hazards model.

The relationship between miR-211 expression and outcome was also evaluated by means of the unsupervised clustering algorithm k-means. This algorithm partitions data points into k groups in an iterative fashion, given a predefined number of clusters k (k = 2, maximum iterations = 1000).

For the Pearson χ^2^ test, Wilcoxon-test, Kaplan-Meier curves, log-rank test and multivariate analysis, data were analyzed using *SPSS v.17* statistical software (SPSS, Inc, Chicago, IL), while all the other computational analyses were performed in *R* (*R v.2.10.1*, packages: stats, dprep). Further details on methods and statistics are provided in the Supplemental data.

### *In vitro* studies

The human PDAC cell lines AsPc-1, Capan-1, CFPAC-1, HPAC, HPAF-II, MIA PaCa-2, PANC-1, PL45, and Su86.86 were purchased from the American Type Culture Collection (ATCC, Manassas, VA), while five primary cell cultures (LPc006, LPc028, LPc033, LPc067, and LPc111) were isolated from patients at the University Hospital of Pisa (Pisa, Italy), as described previously (17). The cells were cultured in RPMI-1640 media, supplemented with 10% FBS, and 1% penicillin (50 IU/mL) and streptomycin (50 µg/mL) (Gibco, Gaithersburg, MD). Cells were kept at 37°C under an atmosphere of 5% CO_2_ in 75 cm^2^ tissue culture flasks (Greiner Bio-One GmbH, Frickenhausen, Germany) and harvested with trypsin-EDTA in their exponentially growing phase. RNA was extracted using a Trizol-chloroform protocol (Sigma, St. Louis, MO). RNA yields and purity were checked by measuring optical density at 260/280 nm with a Nanodrop® spectrophotometer. The basal expression of miR-211 was assessed by qRT-PCR, as described above for PDAC tissues. Amplification data were normalized to RNU6 expression, and quantification of relative expression was performed using the ΔCt method.

The effect of miR-211 on chemosensitivity was evaluated in the MIA PaCa-2 and LPc028 cells, by transfecting these cells with the precursor and antisense oligonucleotides (pre-miR-211 and anti-miR-211) purchased from Ambion-Applied Biosystems (Assay ID, MC10168 and MH10168, respectively) at 30 nM final concentration. Cells were plated at 5,000 cells/well in 200 µl RPMI with 10% FBS and 1% antibiotics. After 24 hours cells were exposed to 0.9 µl oligofectamine (Invitrogen, Paisley, UK) in serum-free medium, mixed for 10 minutes at room temperature, followed by the addition of 0.3 µl of 6.25 µM miR-211 precursor or inhibitor. Cells were also incubated with miRNA negative controls (Ambion). After overnight exposure the medium was removed from the wells and replaced with RPMI with 10% FBS, without antibiotics. Then cells were allowed to grow for additional 48 hours in drug-free medium or treated with 1 µM gemcitabine, as described above. Additional control wells were used for RNA extraction, to evaluate the transfection efficiency.

Finally, in preliminary functional analyses on potential targets of miR-211 predicted by *TargetScan*, we selected ribonucleotide reductase subunit 2 (RRM2), which is an important cellular target of gemcitabine [25]. Therefore, we performed a RT-PCR analysis of the expression of RRM2 in the cells transfected with pre-miR-211 and anti-miR-211, as described above. These PCR reactions were performed with primers and probe from the Applied Biosystems Assay-on-Demand Gene expression product Hs0035724, using a previously validated method [15]. Amplifications were normalized to GAPDH, and quantitation of gene expression was performed using the ΔΔCT calculation, where CT is the threshold cycle; the amount of target gene, normalized to GAPDH and relative to the calibrator (untreated control cells), is given as 2^−ΔΔCT^. Specimens were amplified in triplicate with appropriate non-template controls, and the coefficient of variation was <1% for all replicates.

## Results

### Characteristics of the patients

Table 1 summarizes the clinicopathological characteristics of all the PDAC patients evaluated in the present study. Most patients had stage-T3 grade-2 tumors, with positive lymph nodes, and perineural invasion.

The miRNA microarray analysis was performed in 19 samples that passed the RNA quality criterion. These patients included 13 patients with OS shorter than 1 year (median OS, 8.0, 95% CI, 5.4–10.6) and 6 patients who survived more than 30 months (median OS, 31.0, 95% CI, 30.6–31.4). The Kaplan-Meier plots of these groups are reported in the Figure S2.

A further group of 60 patients was used as a validation cohort, with median OS and DFS of 20.9 and 11.9 months, respectively (See Figure S3 for the Kaplan-Meier plots). The event-rate was 66.7%, and the median follow-up for surviving patients was 21.4 months. In this cohort OS was significantly longer (p = 0.009) for patients harbouring grade 1/2 tumors (median OS, 25.2, 95%CI, 14.7–35.7) than patients with grade 3 PDACs (median OS, 14.8, 95%CI, 11.3–18.3) Data on outcomes according to patients' characteristics are reported in Table S1.

### MiRNA microarray analysis: overall clustering

**Figure 1:**
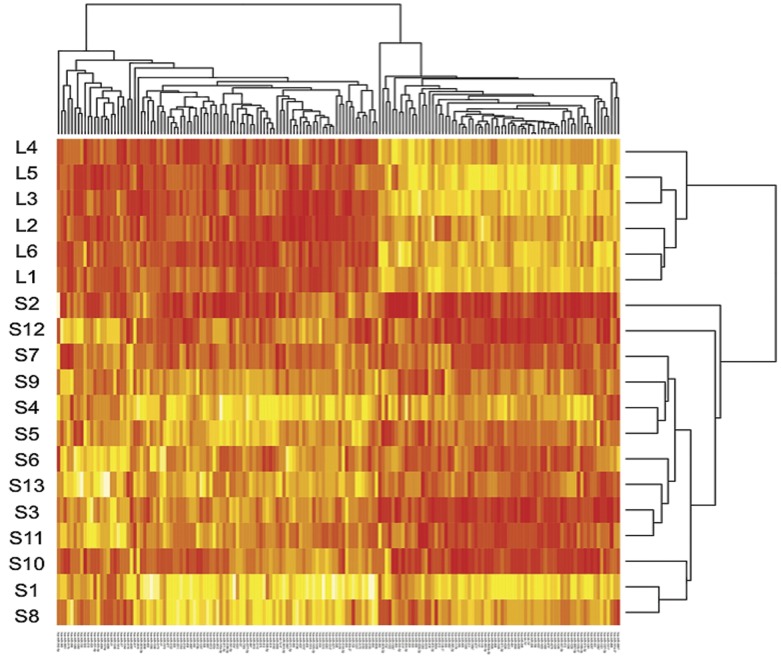
Heatmap of the clustering of 170 miRs filtered based on t-test p-value<0.05. In order to perform hierarchical cluster analysis we constructed a distance matrix using the two-tailed Spearman correlation test, due to the non-normal distribution of expression within samples. The cluster analysis shows a good separation between the two groups of samples, based on the significantly different miRNAs. The microRNA expression data were centered by 2 directions (i.e., by miRNA and patients). Red and yellow represent low and high miRNA expression, respectively.

After the analytical procedures for the normalization of the raw data from the microarray analysis (described in Description S1) we performed a t-test analysis, which resulted in a list of 170 miRNAs that show significant differences in expression between the two groups (p<0.05) (Table S2). In order to perform hierarchical cluster analysis we subsequently constructed a distance matrix using the two-tailed Spearman correlation test, due to the non-normal distribution of expression within samples. This cluster analysis showed a good separation between the two groups of samples (short-OS vs. long-OS), based on the significantly different miRNAs (Figure 1).

### MiRNA microarray analysis: RELIEF and iterative RELIEF

The RELIEF algorithm was employed on the complete data set, as decribed in the methods. This algorithm assigned scores to each miRNA according to how well it discriminated the two groups of samples (e.g., samples from short-OS *versus* samples from long-OS patients). This resulted in a top 10 of most discriminating miRNAs. Figure S3 shows the cluster analysis based on those 10 miRNAs, whilst Table 2 shows this group of top 10 miRNAs and their assigned scores.

**Table 2:** Top-10 most discriminative miRNAs, based on RELIEF score.

Number	miRNA	RELIEF score	Iterative score
1	miR-211	0.372	86
2	miR-4321	0.332	76
3	miR-1207-3p	0.330	81
4	miR-326	0.321	78
5	miR-1914*	0.297	53
6	miR-3610	0.269	32
7	miR-197	0.260	7
8	let-7b*	0.253	43
9	miR-1296	0.250	29
10	miR-4290	0.248	34

After observing how the scores were distributed (Figure S4), we selected the first four (miR-211, miR-4321, miR-1207-3p and miR-326) among this top 10 and performed a cluster analysis.

**Figure 2:**
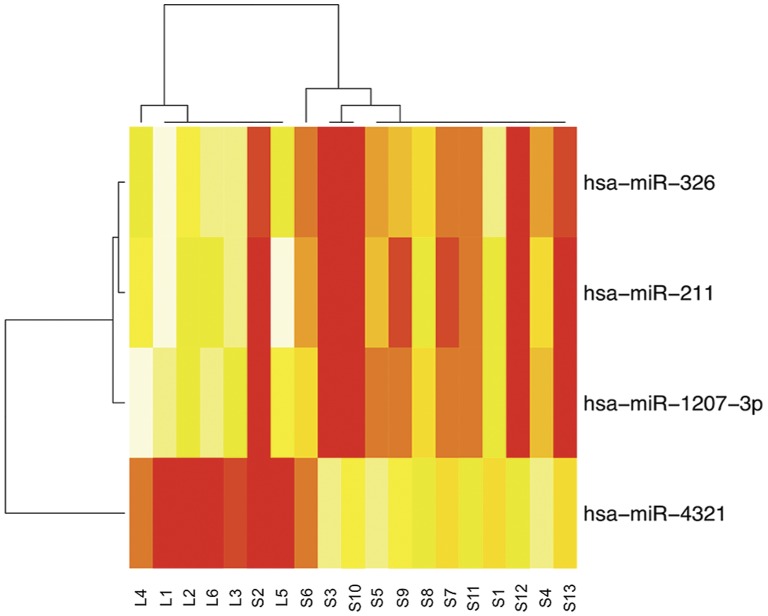
Cluster analysis based on the top 4 most discriminative miRNAs performed with the RELIEF algorithm. The colors are normalized and can only be compared left to right.

As shown in the Figure 2, this top 4 miRNAs clearly separated the two groups of patients. With the exception of one case (S2), which showed very high expression values for all the studied miRNAs, the two main clusters on the x-axis correspond to the two groups (short/long-OS). In particular, since the colors in the heatmap showed the relative expression of the miRNA across all samples, we observed two types of expression profiles, one in which the expression was lower in the patients with a short-OS (for miR-211, miR-1207-3p, miR-326) and one in which the pattern was opposite (for miR-4321). Conversely, patients with long-OS had higher expression values for miR-211, miR-1207-3p, miR-326, and lower expression values for miR-4321.

In order to confirm the most discriminative miRNAs we performed an additional analysis using iterative RELIEF. As observed from Table S3, the top 10 miRs were the same, except for miR-1200 and miR-766 that replaced miR-197 and miR-1296. However, the ranking of the iterative RELIEF scores showed a clearer separation between the top 4 and the bottom 6 most discriminative miRNAs (Figure S6). In this way we demonstrated that the top 4 miRNAs were not biased towards any specific sample. As reported in the Table 2, the miRNAs that appeared in the top 4 using the RELIEF approach also appeared in the top 4 in the iterative RELIEF analysis, suggesting that the expression profile of those 4 miRNAs can be used to confidently distinguish between the patients with short-OS and the patients with long-OS.

### Target prediction for the top miRNA candidates

The identity, chromosomal location and number of target genes of the miRNA candidates identified in our study are summarized in Table 3. To gain further insights into the biological pathways potentially regulated by miRNAs, we next performed a comprehensive comparison between the predicted target genes for our top 4 miRNA candidates according to *TargetScan* and *miRDB*. Importantly, in the *TargetScan* prediction about 10% of these genes were predicted to be targeted by 2 miRNAs, with 13 genes targeted by three miRNA and one gene targeted by all the four different miRNAs (Table S4).

**Table 3:** Identity, chromosomal localization, and number of the predicted target gens of the top-4 most discriminative miRNAs, as predicted with *TargetScan* and *miRDB*.

Number	miRNA	Chromosome	*miRDB* targets*	*TargetScan* targets**
1	miR-211	15q13.3	702	669
2	miR-1207-3p	8q24.21	217	488
3	miR-326	11q13.4	309	441
4	miR-4321	chr19:2250638–2250717°	14	161

Notes:

* *miRDB*, release January, 2012;

** *TargetScan*, release March 2012;

° Coordinates from the Genome Reference Consortium Human genome build 37 (GRCh37).

### Analysis of the prognostic role of miR-211 and miR-4321 in an independent cohort of PDAC patients

RT-PCR analysis of miR-211 expression in 60 independent PDAC samples was used to validate the prognostic significance of this miRNA. This validation group did not differ significantly in terms of clinicopathological characteristics compared to the initial cohort of patients (Table 1).

The expression of miR-211 was detectable in all these samples, and patients were initially categorized according to the median expression value of miR-211 (12.8 a.u.), according to the Gaussian distribution of the expression values, as described in the Figure S7.

Remarkably, miR-211 expression differed significantly between grade 1/2 (N = 30) and grade 3 (N = 30) tumors (P = 0.006 in the Wilcoxon-rank-sum-test). In contrast, no difference was detected in miR-21 expression levels according to other clinicopathological parameters (Table S5).

**Figure 3:**
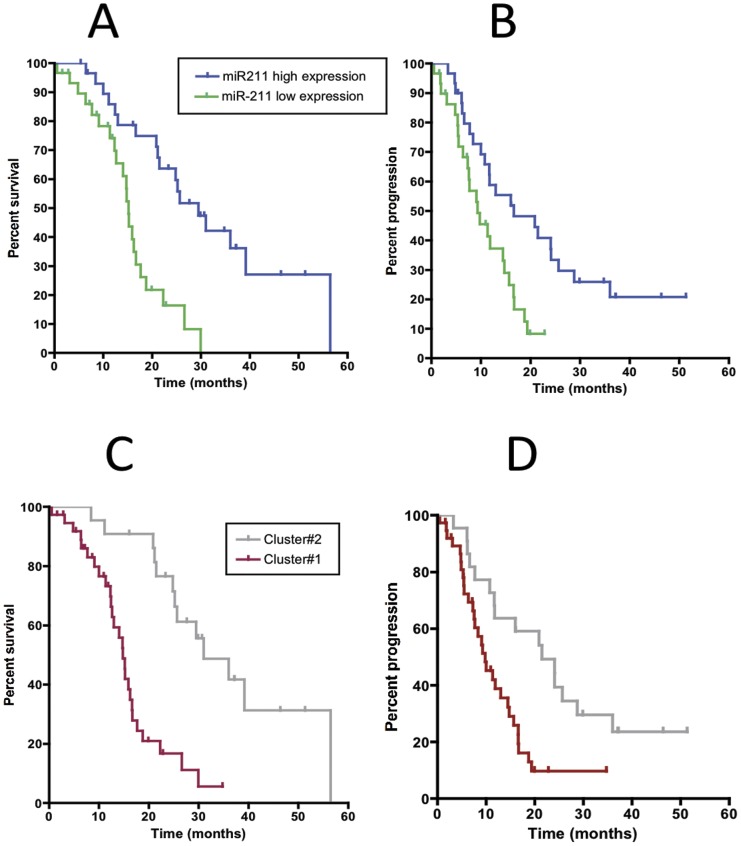
Kaplan-Meier curves for OS and DFS according to (A–B) miR-211 above and below median expression (e.g., low vs. high expression), and to (C–D) k-means clustering.

A strong correlation of miR-211 expression status and clinical outcome was demonstrated. The high miR-211 expression group had a better prognosis than the low expression group. Patients with miR-211 expression below median (low miR-211) had a significantly shorter median OS (14.8 months, 95%CI, 13.1–16.5 months) compared to patients with miR-211 expression higher than median (median OS, 25.7 months, 95%CI, 16.2–35.6 months, HR = 3.0, 95%CI, 2.1–8.9, P<0.001). Similar results were obtained with the DFS curves of patients with miR-211 expression above median, with a median DFS of 16.7, compared to 9.3 months in patients with the lowest miR-211 expression (P = 0.004). The OS and DFS Kaplan-Meier curves are shown in the Figures 3A–B.

Conversely, the expression of miR-4231 was not correlated to outcome in the same cohort of patients (Figure S8). Patients with miR-4231 expression below median had only a trend towards a significant longer median OS compared to patients with miR-4231 expression above median (25.8 months, 95%CI, 18.2–32.3 months, vs. 16.7 months, 95%CI, 13.7–19.7 months, P = 0.194). Similarly, no significant differences were observed for median DFS (13.0 months, 95%CI, 8.4–17.6 months, vs. 10.0 months, 95%CI, 2.0–17.9 months, P = 0.581).

However, given the fact that there was a good correlation between the expression of miR-211 and OS, as shown in the Figure S8 (r = 0.724) we further analyzed the miR-211 expression data using K-means clustering (k = 2). Each of the 60 samples was assigned to one of two clusters (Figure S9), which were compared with the Kaplan-Meier curves for OS and DFS. These analyses showed a significant difference (Figures 3C–D). The median OS of cluster#2 was 31.0 months, 95%CI, 20.0–41.8 months, while median OS of cluster#1 was 14.8 months, 95%CI, 13.3–16.3 months (HR = 3.7, 95%CI, 2.4–9.9, P<0.001). Similarly, the median DFS of cluster#2 was 21.5 months (95%CI, 10.0–33.1 months), while median OS of cluster#1 was 9.8 months (95%CI, 6.5–13.1 months, HR = 2.4, 95%CI, 1.4–2.9, P = 0.002).

To evaluate the risk of disease progression and death we carried out two Cox regression analyses entering all the variables significantly associated with DFS and OS from the univariate model. For the expression values of miR-211, these analyses were performed categorizing patients with the median-value and the K-means clustering.

The Cox proportional hazards regression model used for the multivariate analysis confirmed the prognostic significance of miR-211 expression and grading (Table 4). In particular, low miR-211 expression was significantly associated with an increased risk of death (HR = 2.0, 95%CI, 1.1–4.1, P = 0.04) as well with an increased risk of progression (HR = 2.1, 95%CI, 1.1–4.1, P = 0.02). The multivariate analysis performed with miR-211 clusters confirmed also these data as independently prognostic for both mortality and disease progression (Table 4).

**Table 4:** Factors associated with OS and DFS in the multivariate analysis.

		Multivariate analysis		
Covariates for OS		Hazard ratio (95%CI)	df	*P*
*Grading*	G1–2	0.6 (0.3–1.1)	1	*0.11*
	G3	1 (ref.)		
*miR-211 expression*	Low	2.0 (1.1–4.1)	1	*0.04*
*(vs. median)*	High	1 (ref.)		
*miR-211 expression*	#1	2.8 (1.3–5.8)	1	*0.006*
*(clustering)*	#2	1 (ref.)		
Covariates for DFS		Hazard ratio (95%CI)	df	*P*
*Grading*	G1–2	1 (ref.)	1	*0.04*
	G3	2.23 (1.03–4.85)		
*miR-211 expression*	Low	1 (ref.)	1	*0.24*
*(vs. median)*	High	2.08 (0.61–7.04)		
*miR-211 expression*	#1	2.30 (1.16–4.56)	1	*0.02*
*(clustering)*	#2	1 (ref.)		

Abbreviations: df, degrees of freedom; DFS, Disease Free Survival; OS, Overall Survival.

### Analysis of miR-211 in PDAC cells

**Figure 4:**
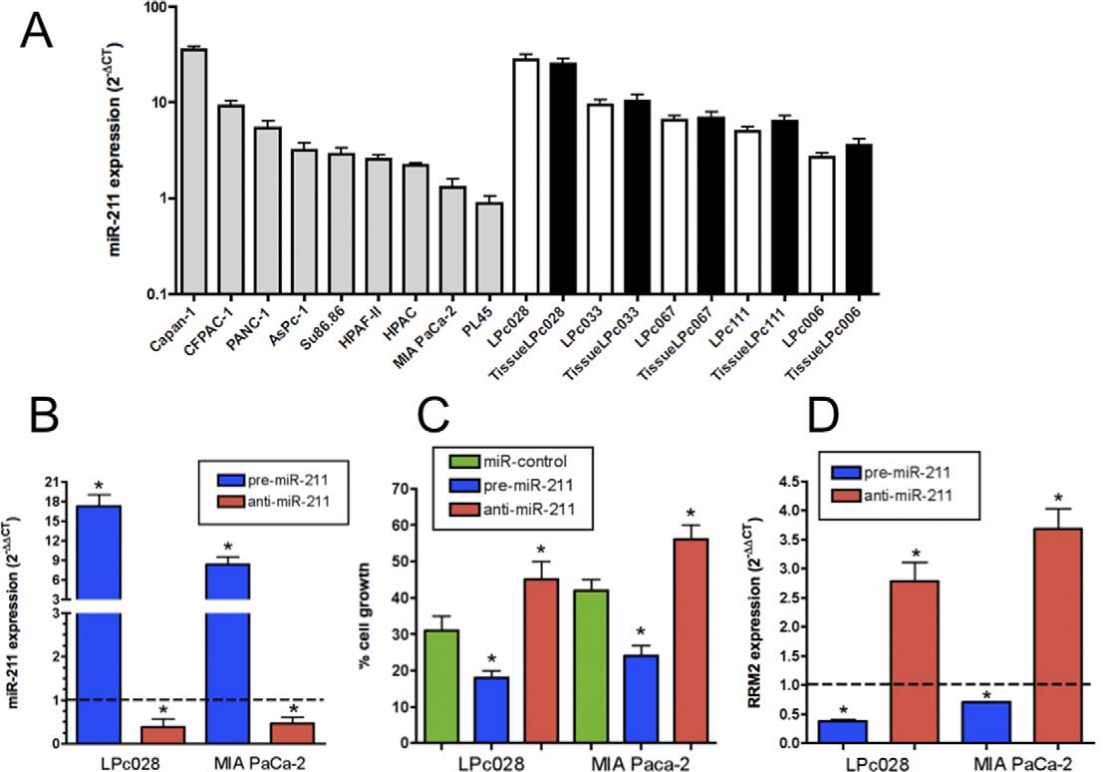
*In vitro* studies validating the role of miR-211 in gemcitabine chemosensitivity. (A) MiR-211 expression in 9 PDAC cell lines and 5 primary tumor cultures. Expression was determined by quantitative PCR, using RNU6 as reference, and the values are in a.u. (B) MiR-211 expression in MIA PaCa-2 and LPc028 cells transfected with negative controls, pre-miR-21 or anti-miR-21 oligos. MiR-211 expression was calculated with the 2^−ΔΔCT^ method with respect to the expression in cells transfected with negative miRNA controls, set as 1, as represented by the dotted line (C) Gemcitabine antiproliferative effects in MIA PaCa-2 and LPc028 cells transfected with miRNA negative controls, pre-miR-21 or anti-miR-21 oligos. *Significantly different from control transfected cells, which were not treated with gemcitabine, set at 100% (P<0.05). (D) RRM2 mRNA expression in MIA PaCa-2 and LPc028 cells transfected with negative controls, pre-miR-21 or anti-miR-21 oligos. RRM2 expression was calculated with the 2^−ΔΔCT^ method with respect to the expression in cells transfected with miRNA negative controls, set as 1, as represented by the dotted line. Columns, mean values obtained from three independent experiments; bars, SE. *Significantly different from control (P<0.05).

The expression of miR-211 was detectable in all the PDAC cell lines and primary tumor cell cultures, ranging from 35.6 in Capan-1 cells to 0.9 a.u. in the PL45 cells (Figure 4A). Of note, miR-211 expression in the 5 primary tumor cells and their originator tumors showed a similar pattern and were highly correlated with Spearman analysis (R^2^ = 0.96, P = 0.01). MIA PaCa-2 and LPc028 cells were selected for further studies because they were representative of cells with very low and very high expression of miR-211, respectively. Transfection efficiency of pre-miR-211 and anti-miR-211 was evaluated by PCR analysis, 24 hours post transfection, showing a significant modulation of miR-211 expression in both cellular models (Figure 4B). In order to evaluate the modulation of gemcitabine anti-proliferative effects, we studied whether treatment with gemcitabine in pre-miR-211 or anti-miR-211 transfected would result in increased/reduced sensitivity compared to cells transfected with miRNA negative controls as well as to cells transfected with miRNA negative controls that were not treated with gemcitabine. As shown in Figure 4C the transfection with pre-miR-211 led to an increased activity of gemcitabine, with a significant reduction of the percentages of cell growth (from 42 to 24% in MIA PaCa-2 cells and from 31 to 18% in LPc028 cells) with respect to cells transfected with miRNA negative controls. Conversely, the transfection with anti-miR-211 caused a significant reduction of the activity of gemcitabine.

Furthermore, we assessed by qRT-PCR if the expression of RRM2 was modulated in the transfected cells, showing its significant reduction in cells with increased expression of miR-211. On the contrary, we observed a significant increase of RRM2 expression in cells with reduced miR-211 expression (Figure 4D).

## Discussion

This is the first study evaluating high-resolution profiles of more than 1200 miRNAs in PDAC patients with homogeneous clinicopathological characteristics, but extremely differential outcome (i.e., short vs. long-OS). Unsupervised hierarchical analysis of the data from this array revealed that PDAC specimens clustered according to their short/long-OS classification.

Highly stringent statistics identified the top 4 discriminating miRNAs (miR-211, miR-1207-3p, miR-326 and miR-4321) between patients with short vs. long-OS, and we performed a validation of the prognostic role of miR-211, which resulted as the best discriminative miRNA and of the rarely investigated miR-4321 in an independent cohort of 60 PDAC. Moreover, we evaluated the role of miR-211 in the modulation of gemcitabine activity in chemosensitivity and PCR studies.

These results support further prospective studies to evaluate the use of miR-211 in risk stratification for resected PDACs.

Although worldwide survival data for PDAC are the lowest among the 60 most frequent types of organ cancers and careful re-evaluation of histological specimens is mandatory in order to avoid misdiagnosis [26], about 20–25% of patients with resectable PDAC survive for more than 5 years after surgery, suggesting that some patients have a less aggressive form of disease [27], [28]. Therefore, the identification of key determinants for the differential aggressiveness of PDAC might be used to stratify patients and guide therapeutic decisions. A multivariate analysis in a case-control study conducted of 357 patients who underwent pancreatoduodenectomy identified lymph node status as the most relevant prognostic factor for long-term survival among clinicopathological characteristics [28]. More recently, a six-gene signature that discriminated optimally between high-risk (aggressive) and low-risk (less aggressive) tumors on the basis of survival, was identified using microarrays analysis of gene expression patterns in primary tumor samples from 15 patients with localized PDAC and 15 patients with metastatic disease [29]. However, the comparison of primary PDAC tumors at the extremes of disease (e.g., early vs. late stage) might not reflect molecular differences in biology within primary PDAC tumors in the same stage, and the search for genes of biological significance in large gene expression datasets is particularly challenging in PDAC.

Therefore, we selected patients with similar clinicopathological characteristics and treatment, but considerable variation in clinical outcomes (e.g., patients with dismal prognosis versus patients who survived more than 30 months). In this unique dataset we performed an array analysis of miRNA, which gave the advantage of investigating multiple regulatory networks likely involved in oncogenic pathways, based on the miRNA ability to target several genes. Another advantage of miRNAs as biomarkers is that technological advances have made it possible to reliably determine their expression using archival FFPE tissues, as also demonstrated by our successful analysis of most samples. This approach is logistically more convenient than evaluating gene expression in frozen tissues, and has relevant implications in studies involving the pancreas, an organ with high endogenous nuclease activity, and a very small amount of tumor tissue available [30].

Several previous studies evaluated detailed large-scale profiles of miRNAs in human PDAC, with a number of studied miRNA ranging from 95 to 866 [31]–[32]. Most studies focused on differences between normal and tumoral pancreatic tissues, or chronic pancreatitis [33]–[42]. The results of these studies have been collected in the *PED database*, which has established itself as one of the main repository for pancreatic-derived -omics data. However, only a few studies evaluated correlation of miRNA profiles with clinical outcome. In particular, Bloomston and colleagues [34], using the miRNA microarray chip OSU_CCC v.3.0, which contains 326 human miRNA probes, identified a subgroup of six miRNAs (miR-30a-3p, miR-105, miR-127, miR-187, miR-452, and miR-518a2) that distinguish long-term survivors with node-positive disease from those succumbing within 24 months. Moreover, grouping the patients according to high or low expression relative to the mean expression of each miRNA on the microarray, two miRNAs were predictive of median survival. High expression of miR-196a-2, which was seen in 75% of tumors, resulted in 2-year survival of 17% compared with 64% for low expression (P = 0.009), whereas median survival in patients with high expression of miR-219 was 13.6 months, compared with 23.8 months for those with low expression, with 2-year survivals of 25% and 49%, respectively (P = 0.07). None of these miRNAs were significantly different in our list of miRNAs filtered based on significant t-test p-value between patients with short/long-OS. Conversely, the recent study of Jamieson and colleagues [43], using Agilent's Human miRNA Microarrays v.2.0, carrying 723 human miRNAs, and a quantitative-PCR method for the validation in a separate cohort of patients, identified the prognostic value of two miRNAs, miR-21 and miR-34a, that were detected among the miRNAs significantly different in our patients with short vs. long-OS. These results are in agreement with several previous studies, supporting the association of high miR-21 expression with poor OS [12]–[13], [44]. Of note, an additional analysis of our previous data in 28 PDAC patients treated with gemcitabine [12], showed that 93% of the patients with high miR-21 expression had also low expression of the most discriminative miRNA emerging from the present study, miR-211. Similarly, 90% of the patients with low miR-21 expression had also high miR-211 expression. Therefore, the survival curves of these miRNAs had a similar shape (Figure S10).

The proposed oncogenic properties of miR-21 are supported by its almost ubiquitously expression as well as by several functional investigations showing that modulation of this miRNA affected proliferation, invasion and chemosensitivity of cancer cell lines, including PDAC cells [45]–[47]. Similarly, preclinical studies in PDAC cells showed that miR-34 was involved in the reversal of the tumor suppressing function of p53 in p53-deficient cells, as well as in pancreatic cancer stem cell self-renewal, potentially via the direct modulation of downstream targets Bcl-2 and Notch [48].

However, to our knowledge, this is the first study unraveling the possible prognostic role of miR-211, miR-1207-3p, miR-326 and miR-4321 in PDAC. MiR-211 is encoded within the sixth intron of *TRPM1*, a candidate suppressor of melanoma metastasis [49]–[50], and previous researches demonstrated that overexpression of miR-211 inhibited both anchorage-independent colony formation and invasion, through regulation of IGF2R, TGFBR2, NFAT5 and BRN2 [51]–[52]. In contrast, a recent study in the colorectal cancer cell line HCT-116 showed that miR-211 expression promotes cellular growth *in vitro* and *in vivo* by targeting the tumor suppressor CHD5 [53], while another study detected an association between higher miR-211 expression and the most advanced nodal metastasis, vascular invasion, and poor prognosis of oral carcinoma [54]. Finally, a recent TaqMan miRNA array for 365 miRNAs identified 24 miRNAs whose expression was altered in two gemcitabine resistant pancreatic cancer cell lines, including the miR-211 homolog miR-204 (sharing the same seed sequence with miR-211). The following qRT-PCR analyses showed that radically resected PDAC patients with high miR-204 expression had significantly longer survival times than those with low expression (P = 0.0054) expression [55]. However this association was observed only in the gemcitabine-treated group, supporting the hypothesis that the prognostic role of mir-204/miR-211 is possibly tumor specific, as well as treatment-related.

Accordingly, in our *in vitro* studies, miR-211 overexpression with a specific pre-miR significantly increased the antiproliferative effects of gemcitabine, while miR-211 suppression caused a significant reduction of gemcitabine activity. Of note, the modulation of miR-211 expression affected the mRNA expression of the predicted target RRM2. RRM2 is a target of gemcitabine activity and a previous study correlated RRM2 expression to gemcitabine sensitivity in PDAC cells [25], supporting the hypothesis that the modulation of gemcitabine sensitivity by miR-211 might be explained at least in part by the modulation of RRM2.

A few studies evaluated the potential role of miR-326 in different cancer types. In particular, this miRNA was downregulated in a panel of advanced breast cancer tissues and reversely associated with expression levels of Multidrug resistance associated protein (MRP-1/ABCC1). Furthermore, elevated levels of miR-326 in the mimics-transfected MCF VP-16-resistant cells reduced MRP-1 expression and sensitized these cells to VP-16 and doxorubicin [56]. Although the impact of ABC transporter family on pharmacokinetics and pharmacodynamics for gemcitabine still remains to be defined, a recent study showed that MRP-1/ABCC1 expression correlated with PDAC tumorigenesis and gemcitabine resistance [57], potentially explaining why our long-survivors patients had a significantly higher expression of its negative regulator miR-326 compared to the patients with short-OS.

Other studies supported the tumor suppressive activity of miRNA-326 through targeting of the Notch pathway in glioma and glioma stem cells [58], as well as its role as suppressor of the pathway activator Smoothened in the regulatory circuitry of the Hedgehog (Hh) signaling, suggesting that alterations of this specific miRNA might sustain cancer development [59]. Notably, the activation of the Hh pathway plays a key role in the desmoplastic hypovascular microenvironment which is now recognized to represent the cardinal histological hallmark feature of PDAC, creating a ‘fortress-like’ hypovascular barrier that impairs the delivery of chemotherapeutics and promotes aggressive neoplastic cell behavior [60]. Therefore, regulation of miR-326 might represent an innovative appealing target to deplete tumor-associated stromal tissue acting on the paracrine signalling axis from neoplastic to stromal cells.

The miR-1207-3p sequence is related to a group of mammalian miRNAs with overlapping seeds (rno-miR-337, mmu-miR-763, and hsa-miR-565) and has been recently detected in a panel of cancer cell lines, during the identification of MiRNAs in the genomically unstable region of human chromosome 8q24 [61] that is also frequently alterated, with high copy number gain, in PDAC [62]. However, no data are available on the possible biological role of this miRNA in cancer. Similarly, no information are available for miR-4321, except that it was identified in the SOLiD ultra-deep sequencing for unique small RNAs from human embryonic stem cells and neural-restricted precursors that were fit to a model of microRNA biogenesis to computationally predict 818 new miRNA genes [63]. However our PCR analysis of miR-4321 expression in an additional cohort of 60 PDAC patients did not confirm its correlation with clinical outcome. These controversial results might be explained by the small sample size, and/or by the hypothesis that miR-4321 could not predict the outcome in an average population of PDAC patients (i.e., a population including both patients with very long/short survival and patients with a survival between 12 and 30 months).

Future functional studies to validate the predicted targets of our top miRNAs are warranted. However, a major drawback for miRNA functional studies is the difficulty in determining the specific target genes regulated by a given miRNA at the transcriptional or translational level. The most commonly used prediction algorithms frequently predict hundreds of target transcripts for any single miRNA, and it is likely that this high number contain a significant fraction of false-positive genes. Therefore, it was not unexpected that the top 4 miRNAs emerging from our study are predicted by *TargetScan* and *miRDB* to potentially be able to target a total of 1575 and 1242 individual transcripts, respectively. In order to reduce this high number and enrich for targets with a potential relevance in PDAC biology, we performed a comparison among the transcripts targeted by multiple miRNAs. This approach reduced the *TargetScan* list of targets to 169 known transcripts, with 13 transcripts targeted by 3 out of our 4 miRNAs. However, it remains difficult to estimate the true false-positive rate of current target prediction algorithms, and the experimental validation of the candidate targets will be an important next step. The list of candidates identified by our enrichment strategy represents, combined with published data, a useful guide for these future studies.

The major strengths of the present study are that it was performed on a homogeneous setting of patients, whose specimens have been all carefully reviewed, and were all treated with the same adjuvant chemotherapy regimen. Moreover, we confirmed the prognostic role of miR-211 in an appropriate validation cohort. Since the Toray's *3D-Gene*™ miRNA chip allowed studying more than 1200 miRNAs, this is the analysis on the largest number of miRNAs ever performed in PDAC. In order to focus on the more discriminative miRNAs we used selective statistical methods with computational analyses performed in the freely available R programming language.

Conversely, the main limitations included the relatively modest sample size of the long-OS patients, and the retrospective explorative single-arm study design. Further studies should validate our candidate miRNAs in a larger cohort, ideally in the prospective and multicentre setting. However, the planning of randomized studies with a control arm of patients treated with other regimens and the comparison of the survival stratified by miRNA expression would be the only way to establish their predictive role. These studies should also establish the potential use of our candidate miRNAs in the neoadjuvant setting, which might provide an excellent alternative for patients with very aggressive disease, who could be given chemotherapy before surgery to kill any micrometastases. Moreover, to overcome the problems regarding tissue availability in the different clinical settings, more accessible samples sources, such as miRNAs enriched tumor derived exosomes in peripheral blood, should be investigated.

In conclusion, our data provide a strong rationale for future mechanistic and clinical studies seeking to link prognostically significant miRNAs, such as miR-211, for their utility as predictive biomarkers and possible innovative tools for molecular therapies in the subset of PDAC patients that they define.

## Supporting Information

Description S1 **Description of the analysis of the raw data from the microRNA array.**
(DOCX)

Figure S1 **Example of PDAC epithelium before (A) and after (B) laser-assisted microdissection, H&E staining of 10 µm thick sections, original magnification, ×10.**
(PPT)

Figure S2 **Kaplan-Meier of OS in (A) the 26 short-OS and long-OS PDAC patients enrolled in this study, and in (B) the 19 patients whose samples were used for the miRNA expression profiling with the Toray's 3D-Gene™ chips.** Event rate was 100%. Statistical differences were analyzed using the log-rank test, as described in the Methods.
(PPT)

Figure S3 **Kaplan-Meier of OS (A) and DFS (B) in the validation cohort of PDAC patients enrolled in this study.**
(PPT)

Figure S4 **Cluster analysis based on the top-10 most discriminative miRNA using RELIEF.** The two main clusters on the x-axis represent the two groups. The colors in the heatmap show the relative expression of the miRNAs across all samples. With the exception of the patient S2, two groups of miRNAs can be observed, one group in which the expression is lower in the patients with short-OS (miR-211, miR-1207-3p, miR-326, miR-197, let-7b*, miR-1296, miR-4290) and one group that has an opposite expression profile (miR-4321, miR-3610, miR-1914*).
(PPT)

Figure S5 **Ranking of the RELIEF scores of top-10 miRNAs.** This ranking was used to select the miRNAs that appeared to be a separate subset.
(PPT)

Figure S6 **Ranking of the iterative RELIEF scores of top-10 miRNAs.** This ranking was used to confirm the 4 most discriminative selected miRNAs.
(PPT)

Figure S7 **Distribution of the expression values of miR-211, evaluated with the R software (“R: A Language and Environment for Statistical Computing”,** http://www.R-project.org **).** The observed Gaussian distributions allowed us to use miR-211 expression data as a dichotomic variable with respect to the median value.
(PPT)

Figure S8 **Kaplan-Meier of OS (A) and DFS (B) according to miR-4321 expression in the validation cohort of PDAC patients.**
(PPT)

Figure S9 **Linear regression between expression of miR-211 and OS and scatter plot showing how the expression of miR-211 in the k-means clustering correlated with OS in the 60 patients used for validation.**
(PPT)

Figure S10 **Kaplan-Meier of OS according to miR-21 (A) and miR-211 (B) expression in 28 PDAC patients treated with gemcitabine in the adjuvant setting, as described previously** [**12**] **.**
(PPT)

Table S1 **Outcome of evaluable patients according to clinical characteristics.**
(DOCX)

Table S2 **List of the miRNAs filtered based on significant t-test p-value between patients with short/long-OS and then used in the overall clustering.** The t-test analysis resulted in a list of 170 miRNAs (ordered alphabetically) that show significant differences in expression between the two groups (p<0.05).
(DOCX)

Table S3 **Top-10 miRs selected using iterative RELIEF.** Eight out of ten miRs in this list also appear in the list obtained using RELIEF.
(DOCX)

Table S4 **List of the transcripts targeted by more than one of the top-4 miRNAs (ordered by number of overlaps and alphabetically within each studied miRNA).**
(DOCX)

Table S5 **Association of miR-211 expression with clinicopathological covariates.**
(DOCX)
