# Supplementary material for: High-Throughput MicroRNA (miRNAs) Arrays Unravel the Prognostic Role of MiR-211 in Pancreatic Cancer
Source: PLoS One. 2012 Nov 14;7(11):e49145. doi: 10.1371/journal.pone.0049145 (PMC3498320; doi:10.1371/journal.pone.0049145)
Supplement: Description S1 — Description of the analysis of the raw data from the microRNA array. (DOCX) [file pone.0049145.s001.docx]

**Description S1.** The signal intensity of all 1212 spots including miRNAs, controls and blanks measured by microarray scanner for all 19 samples assayed were detected in duplicate.

Using the raw data, we identified the background (BG) value which was defined as the mean of the blank spot signal intensity after removing 5% upper and lower outliers, the threshold value which is defined as the BG mean + 2 standard deviation (SD), and the coefficient of variation (CV) of the 532 blank controls for each sample.

The duplicated "BG-subtracted" signal intensities for all 1212 miRNAs were calculated as the raw signal intensities subtracted with the mean BG if the raw signal is more than BG mean +2SD. If one of the duplicated spots shows the signal intensity of less than BG mean +2SD, the remaining spot represents the miRNA. If both of the duplicated spots show the signal intensity of less than mean BG +2SD, the gene is defined as "undetected" and the cells were left blank.

The signal distribution of all samples as well as the mean of each OS group were shown in the included figures.

Since the signal distribution of "BG subtracted" data was similar over samples, the BG subtracted intensities were globally normalized such that the medians of all miRNAs for each sample to be 25.

MiRNAs that had more than one missing data point over all the 19 samples were discarded, while any missing values in the resulting set as well as in the complete data set (N=48) were imputed based on the group average [Supplemental reference#1]. Similarly in the iterative approach all miRNAs that show any missing values over all 12 selected samples were discarded, yielding one hundred sets ranging from 660 to 713 miRs in size.

**Supplemental References**

1. Acuna E, Rodriguez C (2004). The treatment of missing values and its effect in the classifier accuracy. In D. Banks, L. House, F.R. McMorris, P. Arabie, W. Gaul (Eds). Classification, Clustering and Data Mining Applications. Springer-Verlag Berlin-Heidelberg, 639-648
