# Supplementary figures and images for: High-Throughput MicroRNA (miRNAs) Arrays Unravel the Prognostic Role of MiR-211 in Pancreatic Cancer
Source: PLoS One. 2012 Nov 14;7(11):e49145. doi: 10.1371/journal.pone.0049145 (PMC3498320; doi:10.1371/journal.pone.0049145)

## Slide 1
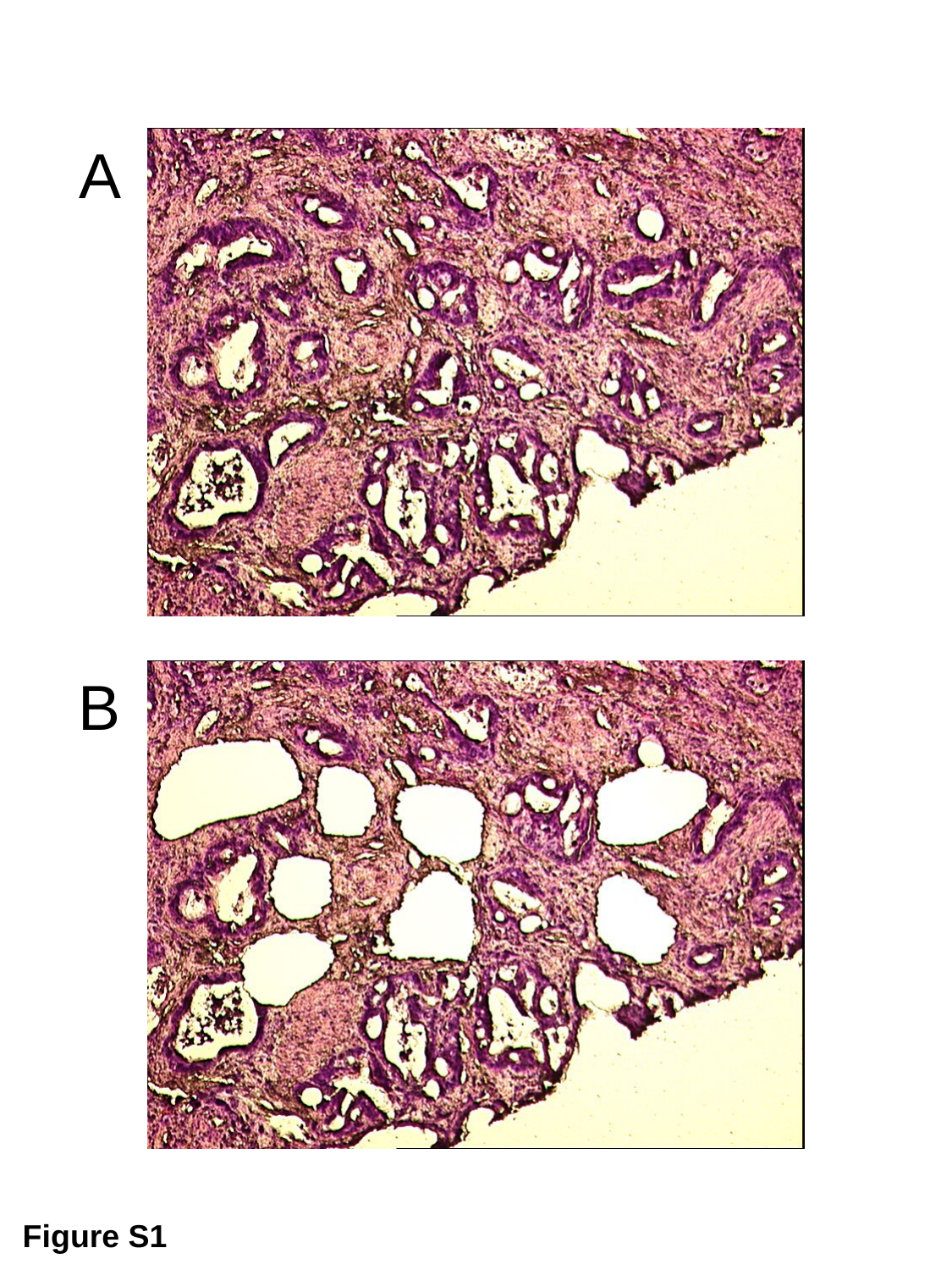

A
B
Figure S1

Supplement: Figure S1 — Example of PDAC epithelium before (A) and after (B) laser-assisted microdissection, H&E staining of 10 µm thick sections, original magnification, ×10. (PPT) [file pone.0049145.s002.ppt]

## Slide 1
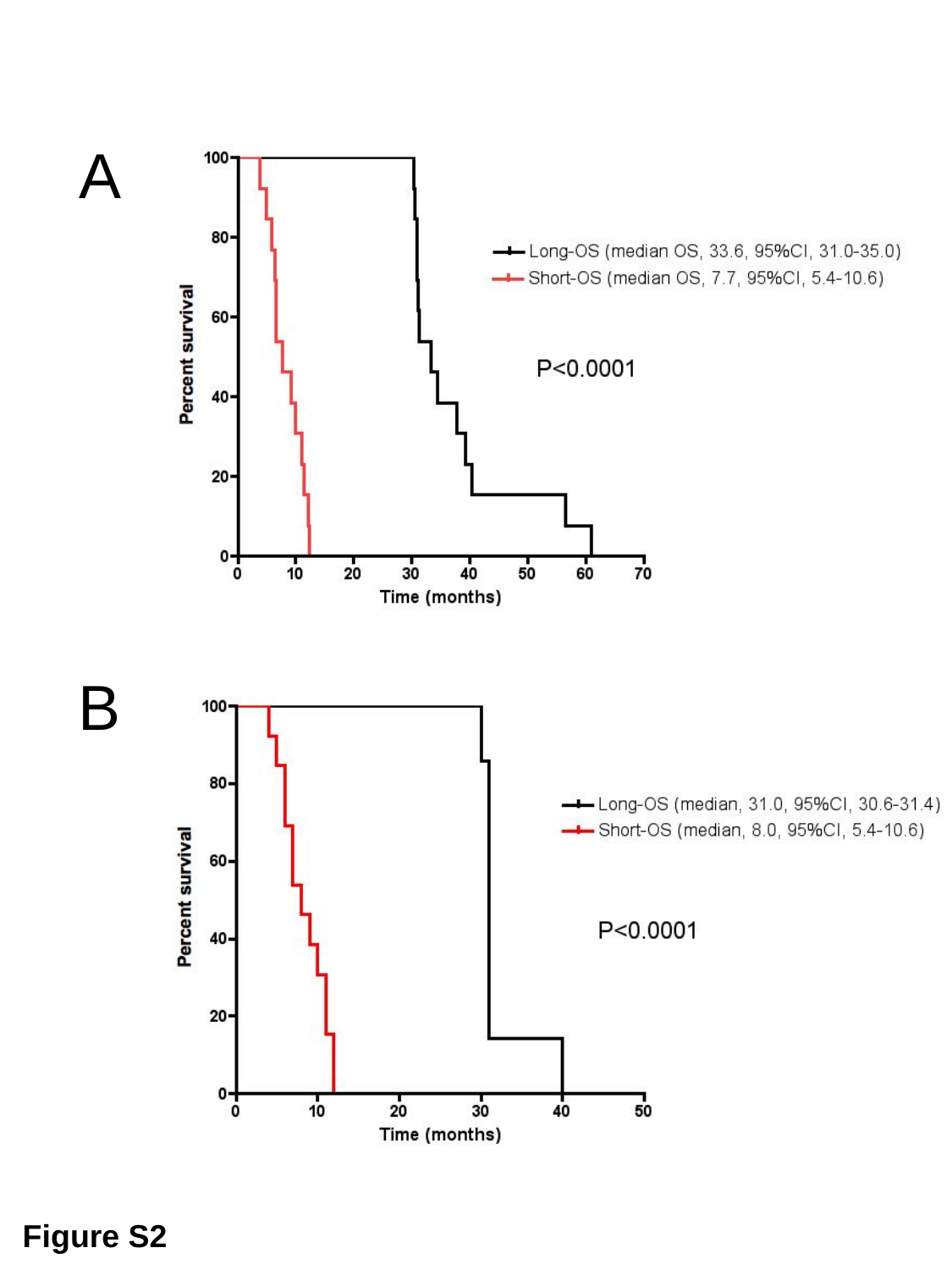

A
B
Figure S2

Supplement: Figure S2 — Kaplan-Meier of OS in (A) the 26 short-OS and long-OS PDAC patients enrolled in this study, and in (B) the 19 patients whose samples were used for the miRNA expression profiling with the Toray's 3D-Gene™ chips. Event rate was 100%. Statistical differences were analyzed using the log-rank test, as described in the Methods. (PPT) [file pone.0049145.s003.ppt]

## Slide 1
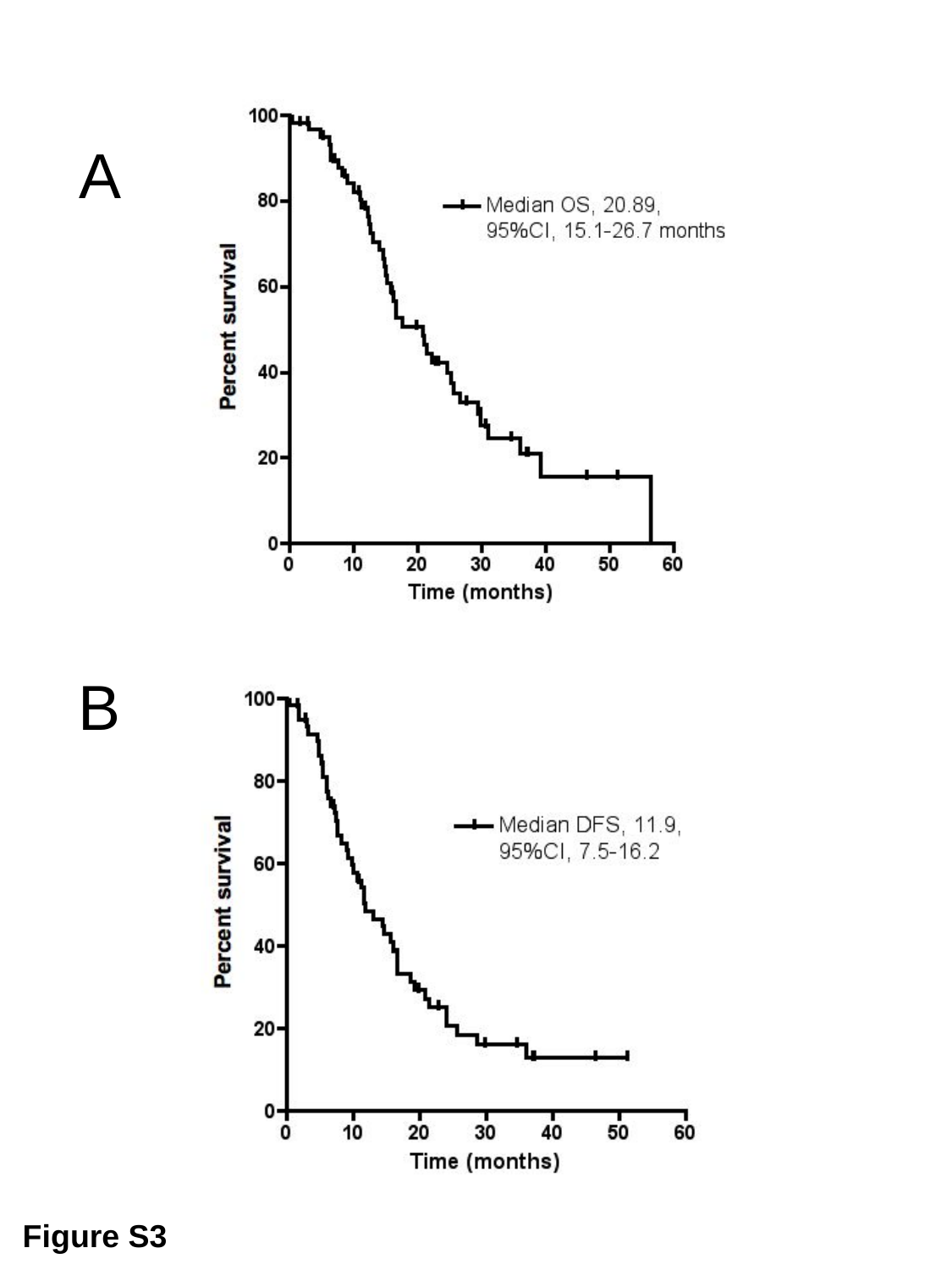

A
B
Figure S3

Supplement: Figure S3 — Kaplan-Meier of OS (A) and DFS (B) in the validation cohort of PDAC patients enrolled in this study. (PPT) [file pone.0049145.s004.ppt]

## Slide 1
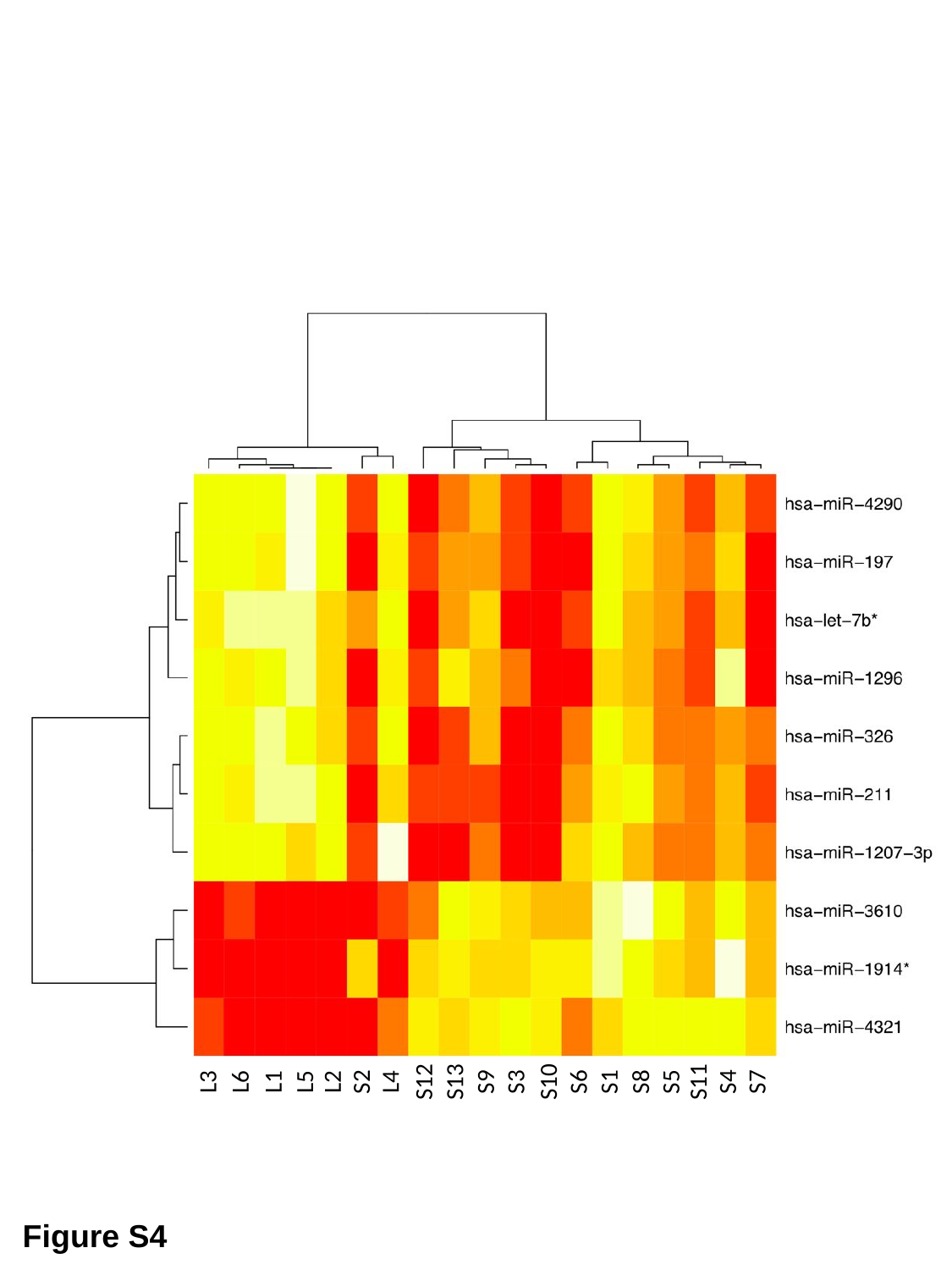

L3
L6
L1
L5
L2
L4
S2
S12
S13
S9
S3
S10
S6
S1
S8
S5
S11
S4
S7
Figure S4

Supplement: Figure S4 — Cluster analysis based on the top-10 most discriminative miRNA using RELIEF. The two main clusters on the x-axis represent the two groups. The colors in the heatmap show the relative expression of the miRNAs across all samples. With the exception of the patient S2, two groups of miRNAs can be observed, one group in which the expression is lower in the patients with short-OS (miR-211, miR-1207-3p, miR-326, miR-197, let-7b*, miR-1296, miR-4290) and one group that has an opposite expression profile (miR-4321, miR-3610, miR-1914*). (PPT) [file pone.0049145.s005.ppt]

## Slide 1
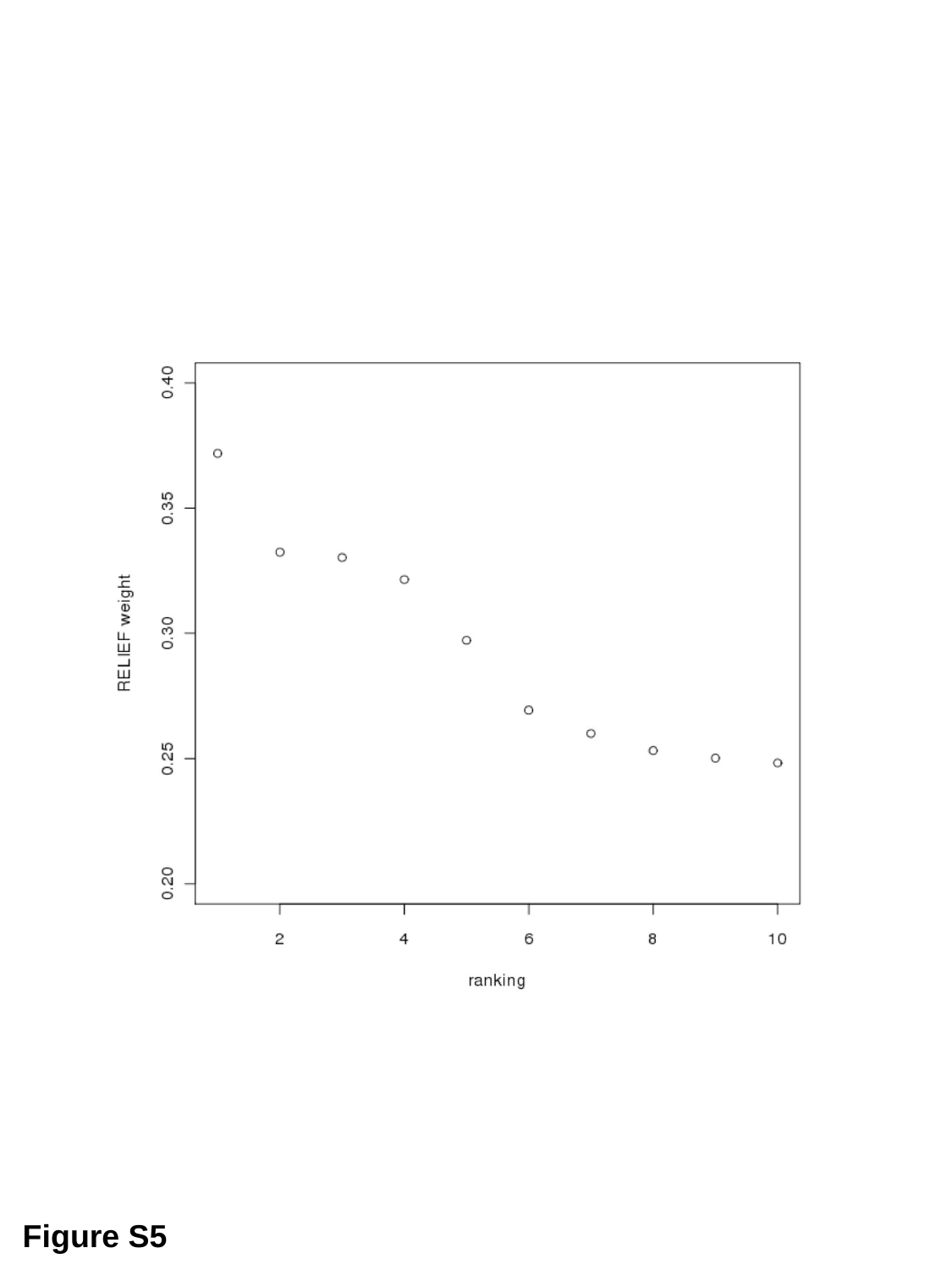

Figure S5

Supplement: Figure S5 — Ranking of the RELIEF scores of top-10 miRNAs. This ranking was used to select the miRNAs that appeared to be a separate subset. (PPT) [file pone.0049145.s006.ppt]

## Slide 1
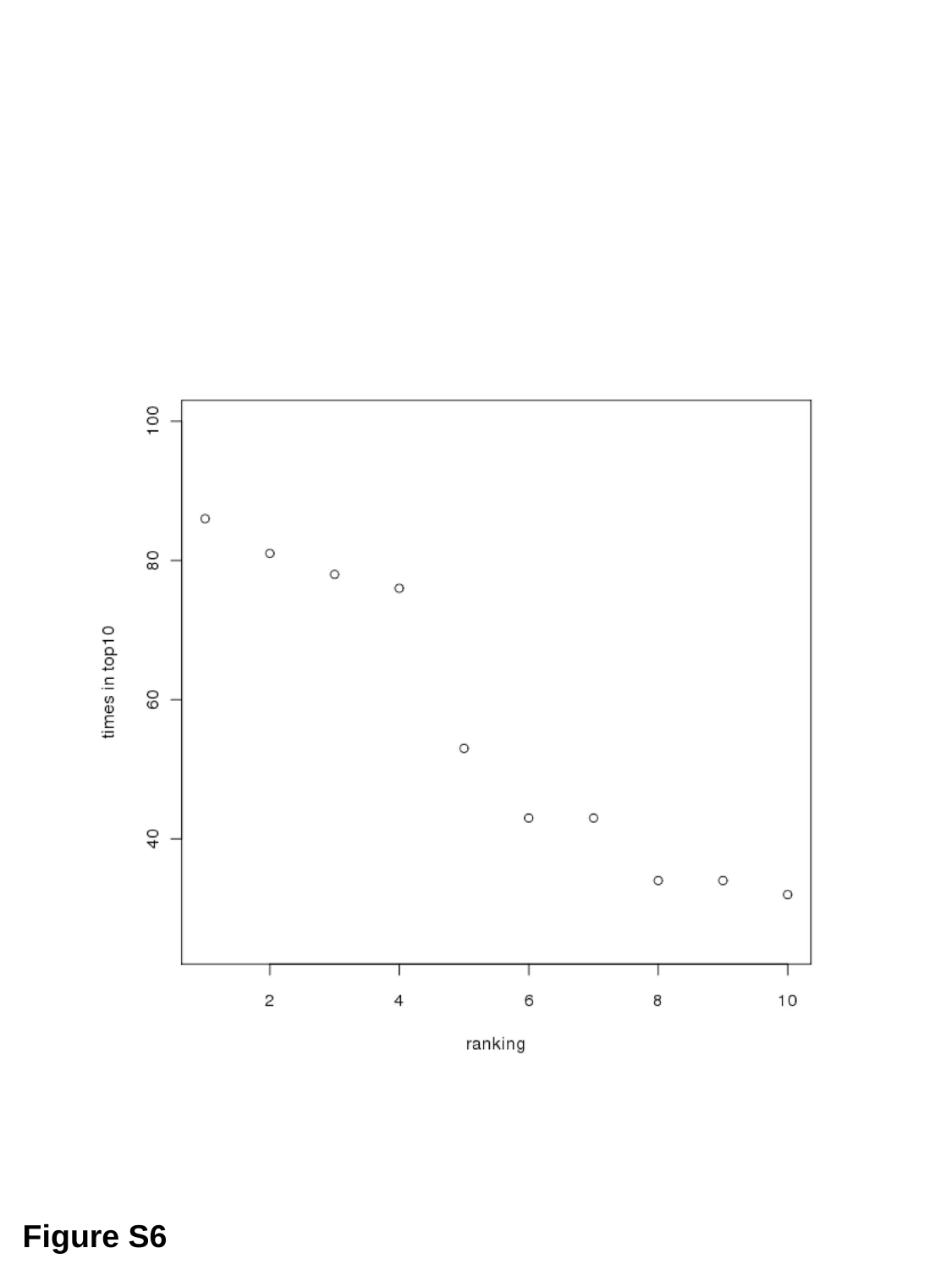

Figure S6

Supplement: Figure S6 — Ranking of the iterative RELIEF scores of top-10 miRNAs. This ranking was used to confirm the 4 most discriminative selected miRNAs. (PPT) [file pone.0049145.s007.ppt]

## Slide 1
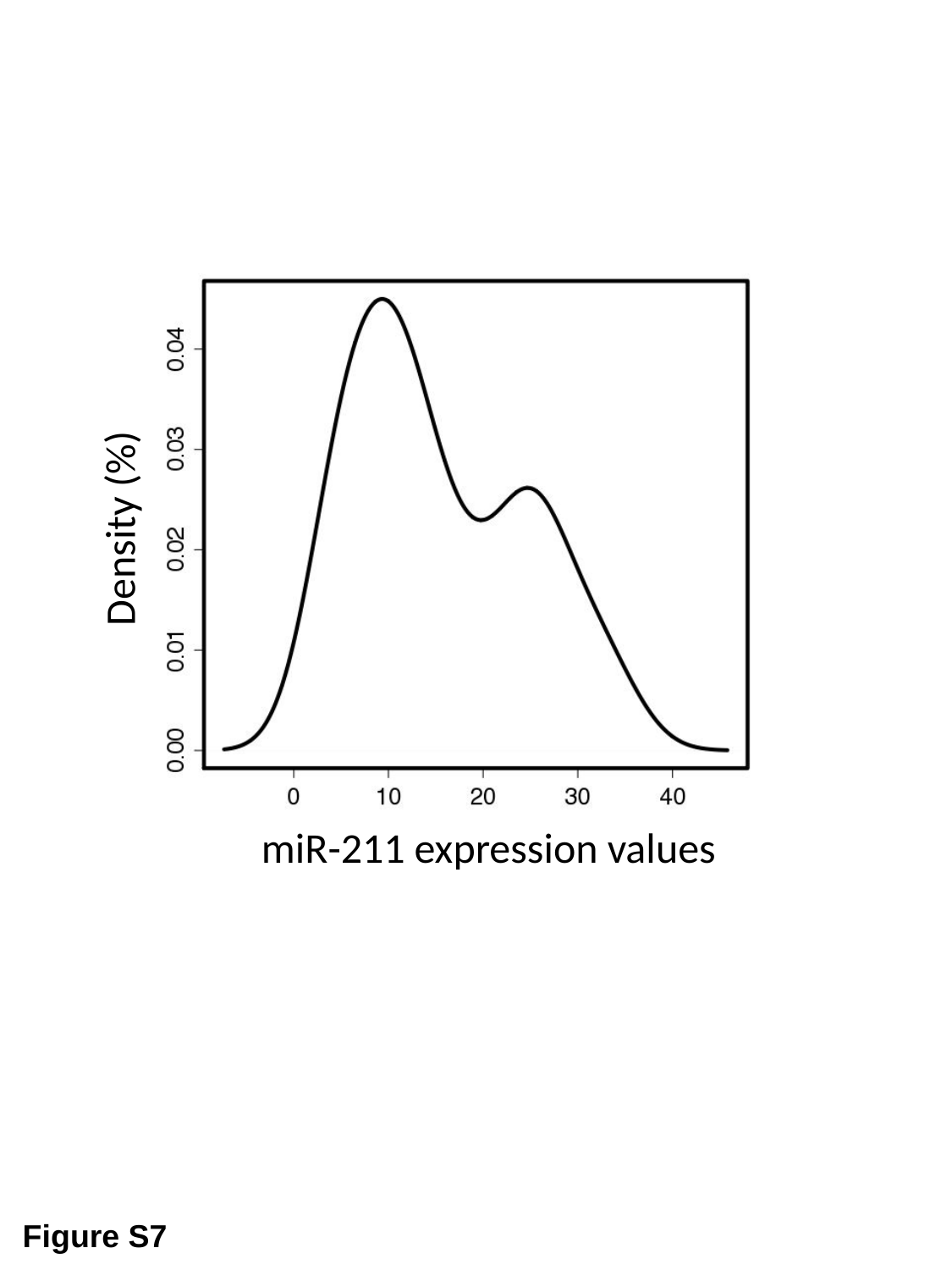

Density (%)
miR-211 expression values
Figure S7

Supplement: Figure S7 — Distribution of the expression values of miR-211, evaluated with the R software (“R: A Language and Environment for Statistical Computing”, http://www.R-project.org ). The observed Gaussian distributions allowed us to use miR-211 expression data as a dichotomic variable with respect to the median value. (PPT) [file pone.0049145.s008.ppt]

## Slide 1
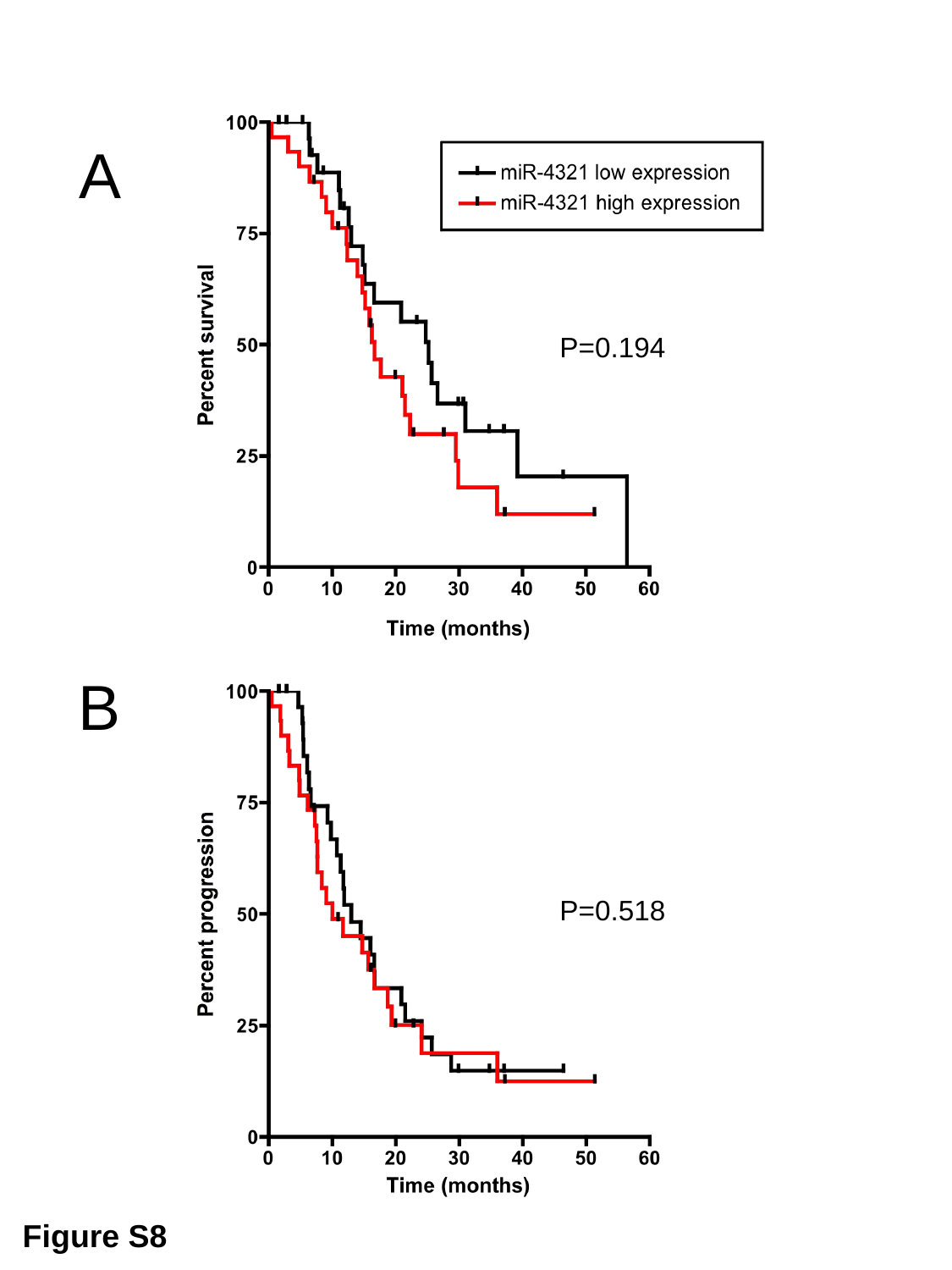

A
P=0.194
B
P=0.518
Figure S8

Supplement: Figure S8 — Kaplan-Meier of OS (A) and DFS (B) according to miR-4321 expression in the validation cohort of PDAC patients. (PPT) [file pone.0049145.s009.ppt]

## Slide 1
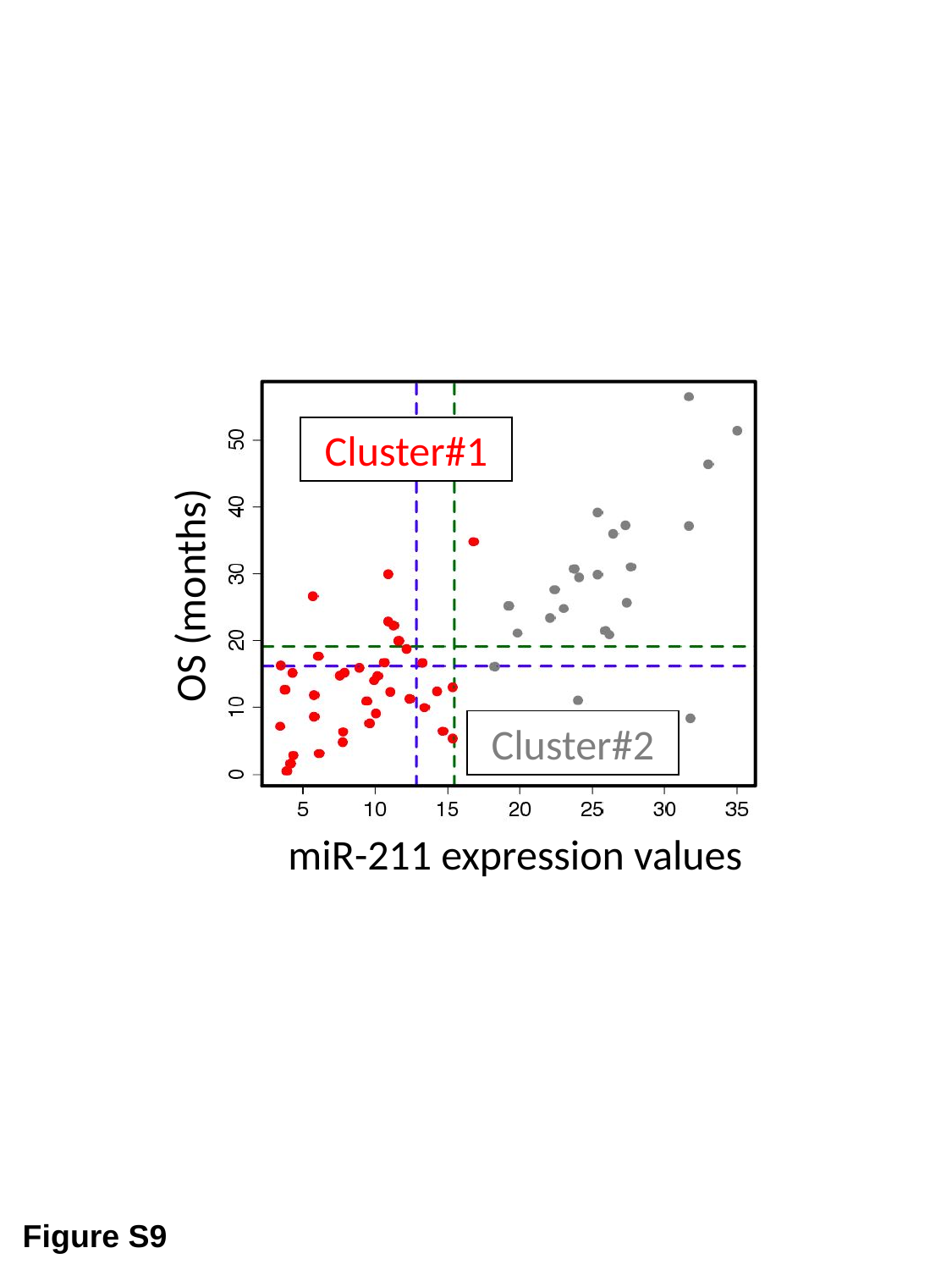

Cluster#1
OS (months)
Cluster#2
miR-211 expression values
Figure S9

Supplement: Figure S9 — Linear regression between expression of miR-211 and OS and scatter plot showing how the expression of miR-211 in the k-means clustering correlated with OS in the 60 patients used for validation. (PPT) [file pone.0049145.s010.ppt]

## Slide 1
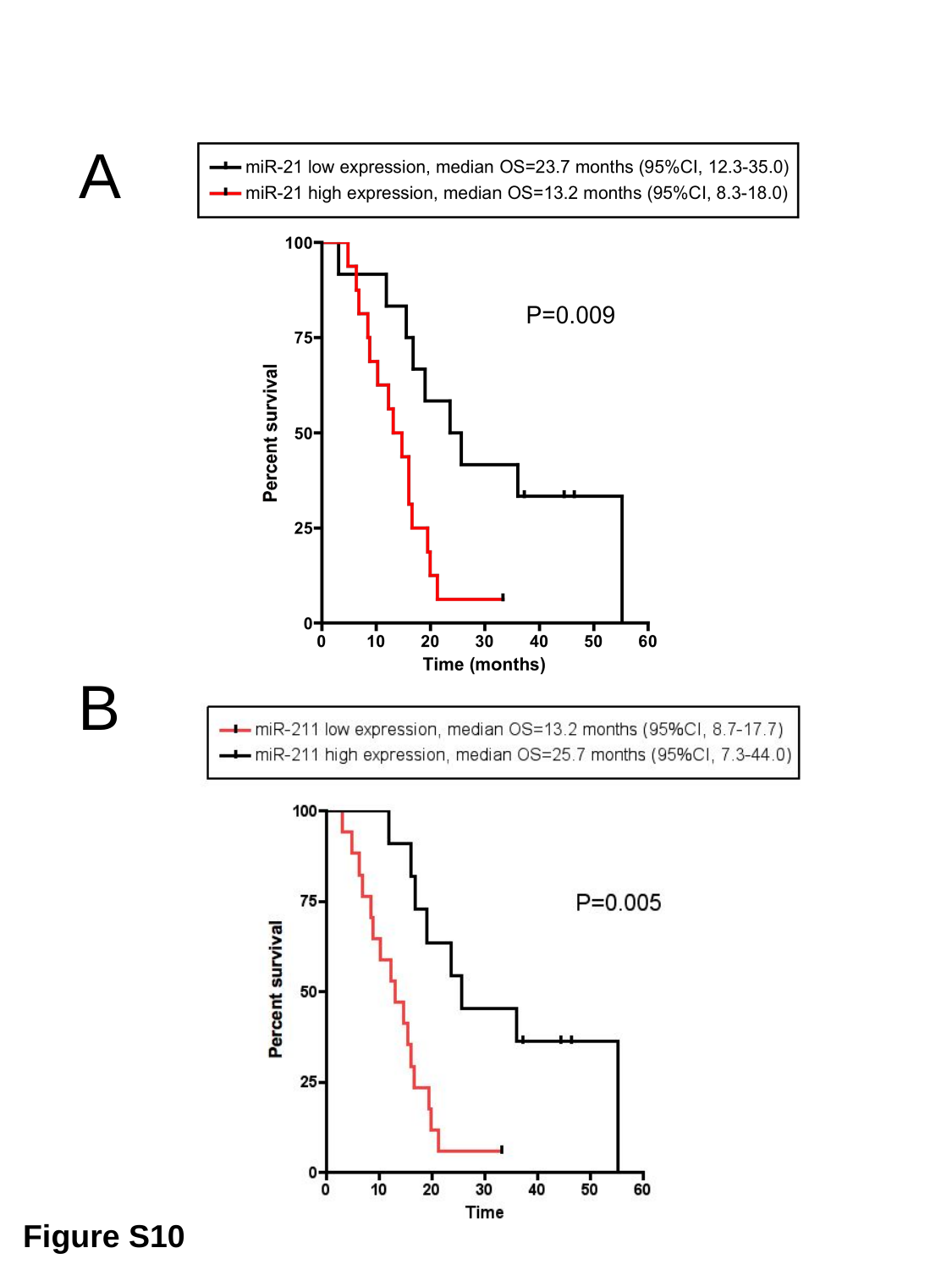

A
B
Figure S10

Supplement: Figure S10 — Kaplan-Meier of OS according to miR-21 (A) and miR-211 (B) expression in 28 PDAC patients treated with gemcitabine in the adjuvant setting, as described previously [12] . (PPT) [file pone.0049145.s011.ppt]
