# Supplementary material for: High-Throughput MicroRNA (miRNAs) Arrays Unravel the Prognostic Role of MiR-211 in Pancreatic Cancer
Source: PLoS One. 2012 Nov 14;7(11):e49145. doi: 10.1371/journal.pone.0049145 (PMC3498320; doi:10.1371/journal.pone.0049145)
Supplement: Table S1 — Outcome of evaluable patients according to clinical characteristics. (DOCX) [file pone.0049145.s012.docx]

| **Table S1.** Outcome of evaluable patients according to clinical characteristics. | | | | | |
| --- | --- | --- | --- | --- | --- |
| **Characteristic** | **Patients**  **n (%)**§ | **mDFS months**  **(95% CI)** | ***P**** | **mOS months**  **(95% CI)** | ***P**** |
| *All* |  |  |  |  |  |
| *Age* |  |  |  |  |  |
| ≤65 years | 36 (60.0) | 11.8 (6.2-17.3) | *0.769* | 17.7 (6.3-29.0) | *0.268* |
| >65 years | 24 (40.0) | 11.9 (7.4-16.4) |  | 21.3 (12.7-30.3) |  |
| *Sex* |  |  |  |  |  |
| Male | 28 (46.7) | 11.7 (7.5-15.2) | *0.490* | 17.7 (6.2-29.1) | *0.837* |
| Female | 32 (53.3) | 13.0 (6.8-16.4) |  | 20.9 (13.2-28.6) |  |
| *Grading* |  |  |  |  |  |
| G1-2 | 30 (50.0) | 19.4 (8.5-30.3) | *0.006* | 25.2 (14.7-35.7) | *0.009* |
| G3 | 30 (50.0) | 9.8 (6.5-13.1) |  | 14.8 (11.3-18.3) |  |
| *Resection margins* |  |  |  |  |  |
| R0 | 52 (90.0) | 13.0 (7.5-18.5) | *0.318* | 20.9 (14.6-27.2) | *0.578* |
| R1 | 8 (13.3) | 11.9 (5.7-18.0) |  | 16.3 (9.8-34.9) |  |
| *Vascular invasion* |  |  |  |  |  |
| Yes | 23 (38.3) | 11.7 (9.2-14.2) | *0.650* | 20.9 (14.4-27.4) | *0.756* |
| No | 37 (31.7) | 14.7 (5.7-23.8) |  | 21.1 (7.9-31.8) |  |
| *Perineural invasion* |  |  |  |  |  |
| Yes | 31 (51.7) | 11.4 (8.2-14.5) | *0.529* | 16.6 (14.3-19.1) | *0.629* |
| No | 29 (48.3) | 14.7 (7.3-22.2) |  | 21.5 (14.4-28.6) |  |
| Abbreviations: mDFS, Median Disease Free Survival; mOS, Median Overall Survival.  § Percentage were calculated with respect to the total number of patients evaluable for the correspondent characteristic.  * *log-rank* test. | | | | | |
