# Supplementary material for: High-Throughput MicroRNA (miRNAs) Arrays Unravel the Prognostic Role of MiR-211 in Pancreatic Cancer
Source: PLoS One. 2012 Nov 14;7(11):e49145. doi: 10.1371/journal.pone.0049145 (PMC3498320; doi:10.1371/journal.pone.0049145)
Supplement: Table S2 — List of the miRNAs filtered based on significant t-test p-value between patients with short/long-OS and then used in the overall clustering. The t-test analysis resulted in a list of 170 miRNAs (ordered alphabetically) that show significant differences in expression between the two groups (p<0.05). (DOCX) [file pone.0049145.s013.docx]

| **Table S2.** List of the miRNAs filtered based on significant t-test p-value between patients with short/long-OS and then used in the overall clustering. The t-test analysis resulted in a list of 170 miRNAs (ordered alphabetically) that show significant differences in expression between the two groups (p < 0.05). | | | |
| --- | --- | --- | --- |
| **miRNA** | **Short mean** | **Long mean** | ***P*** |
| let-7a-2* | 43.55 | 35.17 | 0.01233 |
| let-7b* | 36.914 | 47.767 | 0.00008 |
| let-7d* | 43.987 | 55.484 | 0.00232 |
| miR-15a* | 9.885 | 12.694 | 0.03384 |
| miR-21 | 286.861 | 95.418 | 0.01148 |
| miR-21* | 28.282 | 18.207 | 0.00701 |
| miR-23a* | 27.316 | 19.898 | 0.01112 |
| miR-25* | 109.689 | 71.432 | 0.00037 |
| miR-31 | 159.812 | 30.876 | 0.00287 |
| miR-34b | 21.72 | 27.973 | 0.01585 |
| miR-92a-2* | 280.701 | 204.317 | 0.00657 |
| miR-99b* | 32.942 | 38.067 | 0.01925 |
| miR-106b* | 25.172 | 29.711 | 0.01994 |
| miR-125a-3p | 382.375 | 321.004 | 0.04539 |
| miR-125b-2* | 26.542 | 30.816 | 0.0306 |
| miR-130b* | 19.122 | 22.932 | 0.01376 |
| miR-138-1* | 26.312 | 31.007 | 0.00546 |
| miR-146b-3p | 26.194 | 33.001 | 0.02561 |
| miR-149 | 72.843 | 94.133 | 0.00922 |
| miR-181a-2* | 17.181 | 22.941 | 0.00933 |
| miR-181d | 23.65 | 16.1 | 0.04091 |
| miR-196b* | 14.877 | 19.7 | 0.04708 |
| miR-197 | 88.252 | 129.612 | 0.0001 |
| miR-204 | 20.193 | 29.721 | 0.01338 |
| miR-210 | 142.905 | 79.899 | 0.01334 |
| miR-211 | 35.822 | 51.998 | 0.00007 |
| miR-212 | 24.211 | 29.015 | 0.00643 |
| miR-214* | 34.732 | 45.678 | 0.00516 |
| miR-222 | 210.114 | 126.468 | 0.02573 |
| miR-298 | 18.668 | 14.826 | 0.02289 |
| miR-326 | 56.118 | 78.689 | 0.00001 |
| miR-328 | 126.21 | 167.945 | 0.00049 |
| miR-331-3p | 37.262 | 25.901 | 0.01178 |
| miR-337-3p | 20.471 | 24.221 | 0.00676 |
| miR-363 | 10.103 | 13.131 | 0.01332 |
| miR-365 | 17.938 | 13.976 | 0.02219 |
| miR-365* | 38.351 | 32.925 | 0.02697 |
| miR-371-5p | 160.715 | 107.281 | 0.01543 |
| miR-378* | 19.055 | 24.254 | 0.00022 |
| miR-378b | 25.216 | 21.26 | 0.0311 |
| miR-412 | 26.287 | 35.942 | 0.00005 |
| miR-422a | 106.631 | 78.416 | 0.00645 |
| miR-423-3p | 40.902 | 26.242 | 0.00164 |
| miR-425* | 21.223 | 12.184 | 0.01015 |
| miR-432* | 27.7 | 34.941 | 0.00081 |
| miR-466 | 27.489 | 39.528 | 0.00142 |
| miR-483-3p | 83.126 | 117.448 | 0.00024 |
| miR-485-3p | 40.954 | 57.171 | 0.00366 |
| miR-491-5p | 219.062 | 182.211 | 0.01786 |
| miR-492 | 45.589 | 27.435 | 0.02696 |
| miR-503 | 14.497 | 9.615 | 0.04379 |
| miR-506 | 22.481 | 16.736 | 0.01381 |
| miR-508-5p | 42.082 | 24.836 | 0.01932 |
| miR-517b | 14.791 | 10.282 | 0.01156 |
| miR-532-5p | 14.183 | 21.329 | 0.00838 |
| miR-542-5p | 55.164 | 45.022 | 0.04451 |
| miR-550b | 34.605 | 41.963 | 0.01663 |
| miR-566 | 13.94 | 10.895 | 0.01978 |
| miR-574-3p | 151.874 | 189.826 | 0.00195 |
| miR-591 | 12.454 | 16.269 | 0.0417 |
| miR-593 | 36.571 | 46.034 | 0.01249 |
| miR-595 | 47.593 | 52.772 | 0.04937 |
| miR-602 | 28.402 | 21.762 | 0.00262 |
| miR-605 | 22.921 | 28.494 | 0.00889 |
| miR-612 | 463.099 | 356.223 | 0.04695 |
| miR-614 | 352.283 | 264.983 | 0.00318 |
| miR-617 | 11.003 | 7.605 | 0.02663 |
| miR-654-3p | 14.668 | 18.729 | 0.01116 |
| miR-658 | 268.567 | 216.922 | 0.03368 |
| miR-661 | 29.31 | 36.541 | 0.02316 |
| miR-663 | 1976.986 | 1474.193 | 0.04693 |
| miR-663b | 74.272 | 53.985 | 0.00321 |
| miR-664 | 70.27 | 82.037 | 0.03703 |
| miR-665 | 252.931 | 147.929 | 0.00009 |
| miR-668 | 63.719 | 79.612 | 0.01455 |
| miR-671-3p | 41.482 | 50.184 | 0.0003 |
| miR-671-5p | 431.913 | 377.65 | 0.04867 |
| miR-675 | 339.752 | 271.135 | 0.01939 |
| miR-675* | 95.046 | 111.456 | 0.00379 |
| miR-720 | 3315.652 | 2664.853 | 0.03129 |
| miR-759 | 12.292 | 6.956 | 0.00056 |
| miR-766 | 122.462 | 159.327 | 0.00011 |
| miR-875-3p | 9.492 | 11.995 | 0.03547 |
| miR-877 | 64.56 | 52.25 | 0.01234 |
| miR-877* | 90.1 | 117.753 | 0.00498 |
| miR-887 | 69.505 | 46.491 | 0.00012 |
| miR-888 | 8.374 | 10.67 | 0.02098 |
| miR-892a | 14.378 | 19.511 | 0.01744 |
| miR-920 | 41.021 | 34.522 | 0.04084 |
| miR-934 | 19.292 | 9.854 | 0.00079 |
| miR-935 | 31.537 | 15.134 | 0.00055 |
| miR-940 | 137.398 | 83.042 | 0.00000 |
| miR-1178 | 16.461 | 22.423 | 0.00156 |
| miR-1200 | 30.18 | 40.587 | 0.00038 |
| miR-1207-3p | 31.86 | 42.09 | 0.00002 |
| miR-1225-3p | 44.676 | 30.314 | 0.00011 |
| miR-1226 | 64.766 | 79.051 | 0.00453 |
| miR-1227 | 14.178 | 9.219 | 0.00201 |
| miR-1229 | 126.785 | 160.533 | 0.00923 |
| miR-1236 | 100.203 | 127.896 | 0.00363 |
| miR-1244 | 14.728 | 10.512 | 0.03247 |
| miR-1246 | 2628.859 | 1299.437 | 0.0001 |
| miR-1247 | 26.256 | 20.695 | 0.00523 |
| miR-1248 | 57.948 | 22.716 | 0.00179 |
| miR-1249 | 95.041 | 61.683 | 0.00183 |
| miR-1254 | 55.85 | 46.189 | 0.0236 |
| miR-1260 | 436.684 | 331.149 | 0.0187 |
| miR-1274a | 553.226 | 235.58 | 0.0003 |
| miR-1280 | 3306.884 | 2214.039 | 0.00305 |
| miR-1281 | 134.203 | 179.722 | 0.01675 |
| miR-1284 | 12.208 | 15.329 | 0.01957 |
| miR-1290 | 58.192 | 31.833 | 0.0039 |
| miR-1296 | 51.057 | 63.797 | 0.00043 |
| miR-1303 | 46.712 | 24.33 | 0.00011 |
| miR-1321 | 12.749 | 17.461 | 0.0051 |
| miR-1322 | 15.617 | 19.863 | 0.02866 |
| miR-1468 | 32.886 | 41.671 | 0.00021 |
| miR-1539 | 83.372 | 99.217 | 0.0195 |
| miR-1825 | 91.187 | 127.76 | 0.00193 |
| miR-1911* | 28.46 | 39.036 | 0.00022 |
| miR-1914 | 27.401 | 14.766 | 0.00001 |
| miR-1914* | 233.932 | 90.393 | 0.00000 |
| miR-1915 | 1952.239 | 1014.756 | 0.00033 |
| miR-1915* | 25.661 | 11.666 | 0.00005 |
| miR-1973 | 3235.203 | 2101.11 | 0.0161 |
| miR-2110 | 52.164 | 42.719 | 0.04301 |
| miR-2277-5p | 28.646 | 21.372 | 0.00268 |
| miR-2909 | 12.575 | 9.538 | 0.0374 |
| miR-3116 | 15.155 | 13.133 | 0.04367 |
| miR-3122 | 41.866 | 34.365 | 0.03182 |
| miR-3129 | 11.242 | 7.631 | 0.00382 |
| miR-3144-5p | 14.448 | 11.041 | 0.00547 |
| miR-3154 | 267.03 | 227.682 | 0.00418 |
| miR-3162 | 523.87 | 404.153 | 0.01489 |
| miR-3165 | 13.745 | 9.782 | 0.01382 |
| miR-3173 | 28.104 | 21.034 | 0.01974 |
| miR-3175 | 263.048 | 187.959 | 0.00461 |
| miR-3186-3p | 31.684 | 26.689 | 0.01687 |
| miR-3195 | 505.976 | 426.296 | 0.04219 |
| miR-3196 | 16873.631 | 11532.778 | 0.00159 |
| miR-3200-3p | 11.833 | 16.043 | 0.02574 |
| miR-3610 | 42.927 | 23.908 | 0.00004 |
| miR-3612 | 17.539 | 11.648 | 0.00079 |
| miR-3614-5p | 59.021 | 48.804 | 0.03795 |
| miR-3616-3p | 379.895 | 313.048 | 0.03623 |
| miR-3622a-3p | 68.069 | 84.321 | 0.00341 |
| miR-3648 | 3120.038 | 2385.834 | 0.01665 |
| miR-3651 | 112.235 | 96.16 | 0.02037 |
| miR-3667-3p | 46.384 | 54.716 | 0.02246 |
| miR-3678-5p | 9.802 | 11.678 | 0.03987 |
| miR-3681* | 11.316 | 15.647 | 0.00021 |
| miR-3687 | 744.435 | 481.408 | 0.0101 |
| miR-3907 | 53.477 | 39.03 | 0.00046 |
| miR-3911 | 67.092 | 54.34 | 0.04441 |
| miR-3916 | 9.824 | 6.682 | 0.00142 |
| miR-3940 | 113.45 | 140.711 | 0.00154 |
| miR-4271 | 210.106 | 132.685 | 0.00109 |
| miR-4279 | 129.22 | 168.998 | 0.00262 |
| miR-4281 | 3143.64 | 1706.873 | 0.00005 |
| miR-4284 | 1752.531 | 938.647 | 0.00264 |
| miR-4286 | 3524.511 | 1317.837 | 0.00107 |
| miR-4290 | 94.5 | 143.431 | 0.00001 |
| miR-4292 | 9.951 | 7.446 | 0.01529 |
| miR-4294 | 1080.754 | 894.635 | 0.01954 |
| miR-4297 | 76.365 | 90.983 | 0.02079 |
| miR-4310 | 18.607 | 24.837 | 0.00093 |
| miR-4312 | 50.438 | 62.104 | 0.03694 |
| miR-4313 | 15.115 | 10.825 | 0.01142 |
| miR-4321 | 98.461 | 22.535 | 0.00000 |
| miR-4326 | 55.207 | 66.644 | 0.0065 |
