# Supplementary material for: High-Throughput MicroRNA (miRNAs) Arrays Unravel the Prognostic Role of MiR-211 in Pancreatic Cancer
Source: PLoS One. 2012 Nov 14;7(11):e49145. doi: 10.1371/journal.pone.0049145 (PMC3498320; doi:10.1371/journal.pone.0049145)
Supplement: Table S3 — Top-10 miRs selected using iterative RELIEF. Eight out of ten miRs in this list also appear in the list obtained using RELIEF. (DOCX) [file pone.0049145.s014.docx]

| **Table S3.** Top-10 miRs selected using iterative RELIEF. Eight out of ten miRs in this list also appear in the list obtained using RELIEF. | | |
| --- | --- | --- |
| number | miRNA | score |
| 1 | miR-211 | 86 |
| 2 | miR-1207-3p | 81 |
| 3 | miR-326 | 78 |
| 4 | miR-4321 | 76 |
| 5 | miR-1914* | 53 |
| 6 | let-7b* | 43 |
| 7 | miR-1200 | 43 |
| 8 | miR-4290 | 34 |
| 9 | miR-766 | 34 |
| 10 | miR-3610 | 32 |
