# Supplementary material for: High-Throughput MicroRNA (miRNAs) Arrays Unravel the Prognostic Role of MiR-211 in Pancreatic Cancer
Source: PLoS One. 2012 Nov 14;7(11):e49145. doi: 10.1371/journal.pone.0049145 (PMC3498320; doi:10.1371/journal.pone.0049145)
Supplement: Table S4 — List of the transcripts targeted by more than one of the top-4 miRNAs (ordered by number of overlaps and alphabetically within each studied miRNA). (DOCX) [file pone.0049145.s015.docx]

| **Table S4.** List of the transcripts targeted by more than one of the top-4 miRNAs (ordered by number of overlaps and alphabetically within each studied miRNA) | | | | |
| --- | --- | --- | --- | --- |
| **TranscriptID** | **miR-211** | **miR-1207-3p** | **miR-326** | **miR-4321** |
| NM_000084 | x | x |  |  |
| NM_000175 |  | x | x |  |
| NM_000368 |  | x |  | x |
| NM_000514 | x |  | x |  |
| NM_000555 | x | x |  |  |
| NM_000908 | x | x |  |  |
| NM_000961 | x | x |  |  |
| NM_000966 |  |  | x | x |
| NM_001001669 | x |  | x |  |
| NM_001002860 | x | x |  |  |
| NM_001008220 | x |  | x |  |
| NM_001008701 |  | x | x |  |
| NM_001008781 | x | x |  |  |
| NM_001009899 | x | x |  |  |
| NM_001009993 | x | x |  |  |
| NM_001011666 | x | x |  |  |
| NM_001012393 | x | x |  |  |
| NM_001015051 | x | x |  |  |
| NM_001024843 | x |  |  | x |
| NM_001025076 |  |  | x | x |
| NM_001031695 |  |  | x | x |
| NM_001032280 | x | x |  |  |
| NM_001039360 | x |  | x |  |
| NM_001039469 |  | x | x |  |
| NM_001039570 | x | x |  |  |
| NM_001042599 |  | x | x |  |
| NM_001043318 | x |  |  | x |
| NM_001077397 | x | x |  |  |
| NM_001079526 | x |  |  | x |
| NM_001080410 | x |  | x |  |
| NM_001080420 |  | x |  | x |
| NM_001080744 | x |  | x |  |
| NM_001080779 | x |  | x |  |
| NM_001081491 |  |  | x | x |
| NM_001100422 |  | x | x |  |
| NM_001100626 |  | x |  | x |
| NM_001101372 |  | x | x |  |
| NM_001101802 |  | x | x |  |
| NM_001113178 | x | x |  |  |
| NM_001123066 | x | x |  |  |
| NM_001127192 |  |  | x | x |
| NM_001128159 | x | x |  |  |
| NM_001130059 | x |  |  | x |
| NM_001130916 | x |  |  | x |
| NM_001135825 | x |  | x |  |
| NM_001142281 | x |  | x |  |
| NM_001143888 |  | x | x |  |
| NM_001162429 | x |  | x |  |
| NM_001163438 | x |  |  | x |
| NM_001164093 |  | x | x |  |
| NM_001164749 | x |  | x |  |
| NM_001164766 | x | x |  |  |
| NM_001167671 |  | x | x |  |
| NM_001167738 |  | x | x |  |
| NM_001170765 | x | x |  |  |
| NM_001178091 | x |  |  | x |
| NM_001193288 | x | x |  |  |
| NM_001193304 |  |  | x | x |
| NM_001195278 | x | x |  |  |
| NM_001198625 | x | x |  |  |
| NM_001199427 |  | x | x |  |
| NM_001199839 | x |  | x |  |
| NM_001199880 |  | x | x |  |
| NM_001201366 | x |  |  | x |
| NM_001204144 | x |  |  | x |
| NM_001204458 |  | x | x |  |
| NM_001204848 | x |  | x |  |
| NM_001204856 | x |  | x |  |
| NM_001206491 |  | x | x |  |
| NM_001206957 |  | x | x |  |
| NM_001207025 | x | x |  |  |
| NM_001242314 | x |  |  | x |
| NM_001283 | x |  |  | x |
| NM_001407 | x |  | x |  |
| NM_001830 | x |  | x |  |
| NM_002137 | x |  | x |  |
| NM_002205 |  | x | x |  |
| NM_002373 |  | x | x |  |
| NM_002515 | x | x |  |  |
| NM_002524 |  | x | x |  |
| NM_002609 | x | x |  |  |
| NM_002819 |  | x | x |  |
| NM_002867 |  | x |  | x |
| NM_002868 |  | x | x |  |
| NM_004041 | x | x |  |  |
| NM_004091 |  | x | x |  |
| NM_004232 | x |  |  | x |
| NM_004429 |  | x | x |  |
| NM_004745 |  | x | x |  |
| NM_005105 |  | x |  | x |
| NM_005109 |  | x | x |  |
| NM_005207 | x |  |  | x |
| NM_005215 | x | x |  |  |
| NM_005359 | x | x |  |  |
| NM_005458 | x |  | x |  |
| NM_005633 | x |  | x |  |
| NM_005737 | x | x |  |  |
| NM_005840 | x |  | x |  |
| NM_006078 | x |  |  | x |
| NM_006306 |  | x |  | x |
| NM_006371 | x |  | x |  |
| NM_006690 |  | x | x |  |
| NM_006742 | x |  | x |  |
| NM_006933 |  | x | x |  |
| NM_006965 | x | x |  |  |
| NM_007011 |  | x | x |  |
| NM_007249 | x | x |  |  |
| NM_012306 |  | x | x |  |
| NM_013449 | x |  |  | x |
| NM_014117 | x |  |  | x |
| NM_014232 |  | x | x |  |
| NM_014247 |  | x |  | x |
| NM_014388 |  | x | x |  |
| NM_014598 | x | x |  |  |
| NM_014911 | x | x |  |  |
| NM_015002 |  | x | x |  |
| NM_015143 | x |  | x |  |
| NM_015144 | x |  | x |  |
| NM_015557 | x | x |  |  |
| NM_015630 | x | x |  |  |
| NM_015686 | x |  | x |  |
| NM_016376 | x |  | x |  |
| NM_016531 | x |  | x |  |
| NM_017759 | x |  | x |  |
| NM_018243 | x | x |  |  |
| NM_020336 |  |  | x | x |
| NM_020452 |  |  | x | x |
| NM_020791 | x | x |  |  |
| NM_020949 | x |  | x |  |
| NM_021079 | x | x |  |  |
| NM_021964 | x |  | x |  |
| NM_021974 | x |  | x |  |
| NM_022153 |  | x |  | x |
| NM_024408 | x |  |  | x |
| NM_025074 | x |  | x |  |
| NM_025179 | x | x |  |  |
| NM_025185 | x |  | x |  |
| NM_030761 | x | x |  |  |
| NM_032128 | x | x |  |  |
| NM_032270 | x | x |  |  |
| NM_032575 |  |  | x | x |
| NM_032785 | x |  |  | x |
| NM_033129 | x | x |  |  |
| NM_033133 | x | x |  |  |
| NM_033224 |  | x | x |  |
| NM_033389 |  |  | x | x |
| NM_058172 |  | x |  | x |
| NM_152345 |  | x | x |  |
| NM_152756 | x | x |  |  |
| NM_172250 | x |  | x |  |
| NM_173642 | x | x |  |  |
| NM_178450 | x | x |  |  |
| NM_178815 | x | x |  |  |
| NM_198321 |  | x |  | x |
| NM_198988 |  | x |  | x |
| NM_001080417 | x |  | x | x |
| NM_001083962 | x |  | x | x |
| NM_001134337 | x | x |  | x |
| NM_001160423 |  | x | x | x |
| NM_001204197 | x | x |  | x |
| NM_002745 | x |  | x | x |
| NM_004586 | x | x | x |  |
| NM_004588 | x | x | x |  |
| NM_005811 |  | x | x | x |
| NM_006943 |  | x | x | x |
| NM_012156 |  | x | x | x |
| NM_014631 | x | x | x |  |
| NM_014877 | x |  | x | x |
| NM_024577 | x | x | x | x |
