# Supplementary material for: High-Throughput MicroRNA (miRNAs) Arrays Unravel the Prognostic Role of MiR-211 in Pancreatic Cancer
Source: PLoS One. 2012 Nov 14;7(11):e49145. doi: 10.1371/journal.pone.0049145 (PMC3498320; doi:10.1371/journal.pone.0049145)
Supplement: Table S5 — Association of miR-211 expression with clinicopathological covariates. (DOCX) [file pone.0049145.s016.docx]

| **Table S5.** Association of miR-211 expression with clinicopathological covariates. | | | | |
| --- | --- | --- | --- | --- |
| **Characteristic** | **Low miR-21**  **(%)** | **High miR-211**  **(%)** | **Median miR-211**  **expression** | **P**  ***(Wilcoxon)*** |
| *Age* |  |  |  |  |
| ≤65 years | 14 (38.9) | 16 (72.7) | 15.0 | *0.590* |
| >65 years | 22 (61.1) | 8 (27.3) | 11.0 |  |
| *Sex* |  |  |  |  |
| Male | 16 (53.3) | 12 (40.0) | 11.9 | *0.754* |
| Female | 14 (46.7) | 18 (60.0) | 13.8 |  |
| *Grading* |  |  |  |  |
| G1-2 | 9 (30.0) | 21 (70.0) | 19.5 | *0.003* |
| G3 | 21 (70.0) | 9 (30.0) | 8.9 |  |
| *Resection margins* |  |  |  |  |
| R0 | 23 (76.7) | 29 (96.7) | 14.5 | *0.109* |
| R1 | 7 (23.3) | 1 (3.3) | 5.8 |  |
| *Vascular invasion* |  |  |  |  |
| Yes | 13 (43.3) | 10 (33.3) | 15.3 | *0.366* |
| No | 17 (56.7) | 20 (66.7) | 11.3 |  |
| *Perineural invasion* |  |  |  |  |
| Yes | 18 (60.0) | 13 (43.3) | 10.9 | *0.297* |
| No | 12 (40.0) | 17 (56.7) | 14.7 |  |
| Note: Data on age, sex, resection margins and vascular/perineural invasion were available for all the patients, whilst a total of 59 out of the 60 patients were evaluable for grading. | | | | |
